# DNA methylation as a mediator of genetic and environmental influences on Parkinson’s disease susceptibility: Impacts of alpha-Synuclein, physical activity, and pesticide exposure on the epigenome

**DOI:** 10.3389/fgene.2022.971298

**Published:** 2022-08-19

**Authors:** Samantha L. Schaffner, Michael S. Kobor

**Affiliations:** ^1^ Edwin S. H. Leong Healthy Aging Program, Faculty of Medicine, University of British Columbia, Vancouver, BC, Canada; ^2^ Department of Medical Genetics, British Columbia Children’s Hospital Research Institute, University of British Columbia, Vancouver, BC, Canada

**Keywords:** Parkinson’s disease, neurodegeneration, epigenetics, DNA methylation, gene-environment interaction

## Abstract

Parkinson’s disease (PD) is a neurodegenerative disorder with a complex etiology and increasing prevalence worldwide. As PD is influenced by a combination of genetic and environment/lifestyle factors in approximately 90% of cases, there is increasing interest in identification of the interindividual mechanisms underlying the development of PD as well as actionable lifestyle factors that can influence risk. This narrative review presents an outline of the genetic and environmental factors contributing to PD risk and explores the possible roles of cytosine methylation and hydroxymethylation in the etiology and/or as early-stage biomarkers of PD, with an emphasis on epigenome-wide association studies (EWAS) of PD conducted over the past decade. Specifically, we focused on variants in the *SNCA* gene, exposure to pesticides, and physical activity as key contributors to PD risk. Current research indicates that these factors individually impact the epigenome, particularly at the level of CpG methylation. There is also emerging evidence for interaction effects between genetic and environmental contributions to PD risk, possibly acting across multiple omics layers. We speculated that this may be one reason for the poor replicability of the results of EWAS for PD reported to date. Our goal is to provide direction for future epigenetics studies of PD to build upon existing foundations and leverage large datasets, new technologies, and relevant statistical approaches to further elucidate the etiology of this disease.

## 1 Introduction

Parkinson’s disease (PD) is the second most common neurodegenerative disorder, affecting an estimated 9.4 million individuals worldwide in 2020 (Maserejian et al., 2020). While approximately 5%–10% of PD cases are monogenically inherited, the remaining 90%–95% are considered “sporadic,” with genetics, environment, and gene–environment interactions contributing to varying risk profiles in affected individuals (Lesage and Brice, 2009; Dunn et al., 2019; Pang et al., 2019). One of the genes most closely involved with PD risk is *SNCA*, which encodes alpha-synuclein (α-Syn), a multifunctional protein that is localized at synaptic terminals (Villar-Piqué et al., 2016). Whole-gene multiplications of *SNCA* seen in familial PD and allelic variants in the *SNCA* promoter (REP1) can lead to increased expression of the protein and Lewy body aggregation in dopaminergic neurons (Chiba-Falek and Nussbaum, 2001; Maraganore et al., 2006). Conversely, *SNCA* point mutations can result in loss of function, with consequent impairment of neuronal health. These *SNCA* multiplications and point mutations occur in familial PD, while single nucleotide polymorphisms (SNPs) in *SNCA* can also contribute to risk of sporadic PD (Lesage and Brice, 2009).

Despite the high penetrance of familial PD-associated *SNCA* variants, the reasons underlying incomplete penetrance observed in certain individuals are not well understood. In addition, there is increasing interest in identification of PD biomarkers that could be used to detect disease before onset of symptoms and to prescribe lifestyle interventions to either prevent or slow progression of the disease. Epigenetic marks are attractive candidates for involvement in PD etiology due to their ability to mediate genetic and environmental effects on phenotype, which may partially explain the “missing heritability” in PD (Angelopoulou et al., 2022). The epigenome is comprised of a myriad of factors other than the DNA sequence that influence gene transcription, chromatin interactions, splicing, and other mechanisms. Epigenetic modifications are dynamically influenced by the complex interactions between genotype, environment, lifestyle, and developmental stage (Kumar et al., 2018). DNA methylation (DNAm), which typically involves the addition of a methyl group to the fifth carbon of the pyrimidine ring of cytosine in the CpG dinucleotide context, is one such epigenetic mark that has been extensively characterized in human populations. In addition to its plasticity with respect to genes and environment, DNAm also has potential as a biomarker due to concordance of DNAm patterns between the blood and brain at specific loci (Edgar et al., 2017).

Although epigenome-wide association studies (EWAS) have demonstrated altered DNAm patterns in individuals with PD in comparison to healthy subjects, the majority of findings were not replicable between studies (Masliah et al., 2013; Moore et al., 2014; Henderson-Smith et al., 2019; Li et al., 2020; Marshall et al., 2020; Vallerga et al., 2020; Henderson et al., 2021; Kia et al., 2021; Nabais et al., 2021; Kaut et al., 2022) (Supplementary Tables S1, S2). However, PD-related DNAm changes in particular genes, such as *CYP2E1*, have been reported in brain and blood across multiple studies (Kaut et al., 2012; Henderson-Smith et al., 2019; Kaut et al., 2022). Although few PD EWAS have assessed DNAm patterns in brain and blood from the same individuals, a study in five PD patients and six controls found 124 differentially methylated genes that showed concordant changes in brain and blood, representing 30% of the total annotated genes with differential methylation (Masliah et al., 2013). These observations suggest that blood DNAm in PD could be informative for specific loci in the brain. In addition, the extent to which DNAm patterns are associated with particular genetic backgrounds or environmental/lifestyle exposures in individuals with PD is an area of active research (Dunn et al., 2019; Angelopoulou et al., 2022). Although a number of SNPs associated with PD appear to affect DNAm and some studies have uncovered differential DNAm patterns in PD patients exposed to various drugs or pesticides, how these factors interact to influence risk in undiagnosed individuals remains unclear (International Parkinson’s Disease Genomics Consortium and Wellcome Trust Case Control Consortium, 2011; Nalls et al., 2014; Henderson-Smith et al., 2019; Go et al., 2020; Vallerga et al., 2020). Multi-omics studies including analysis of genotype, gene expression, and/or other epigenetic modifications alongside DNAm will also help to clarify the role of this epigenetic mark in the molecular etiology of PD (Kia et al., 2021).

Accumulating evidence also suggests that other cytosine modifications beyond DNAm have specific functions in the brain, including non-CpG methylation and DNA hydroxymethylation (DNAhm) (Kinde et al., 2015). Research on the role of DNAhm in PD is in its infancy, with initial studies reporting increased DNAhm in PD cerebellar white matter and increases in DNAhm and Ten-Eleven Translocation 2 (*TET2*) expression in purified neurons from PD patients (Kaut et al., 2019; Marshall et al., 2020). Taken together, these findings indicate that analysis of cytosine methylation has great potential for increasing our understanding of the etiologies underlying complex neurological disorders, such as Alzheimer’s disease and PD (Delgado-Morales and Esteller, 2017).

Here, we provide a comprehensive overview of cytosine modifications in PD in the context of *SNCA* genetic background and environmental inputs. We also discuss recent developments in PD biomarker discovery and integrated multi-omics strategies, along with future perspectives for PD epigenetics research.

## 2 Parkinson’s disease

Parkinson’s disease has an estimated prevalence of 4% among adults over 85 years old (Corti et al., 2011; Coppedè, 2012). The disease is caused by the degeneration of dopaminergic neurons in the substantia nigra, across other brain regions including the cortex, hippocampus, and brainstem, and in components of the central and peripheral nervous systems, leading to a range of motor and nonmotor phenotypes, including resting tremor, rigidity, bradykinesia, constipation, depression, and dementia (Lang and Lozano, 1998; Inzelberg et al., 2004). Although several monogenic forms of PD have been identified, up to 95% of PD cases are classified as sporadic, with no previous family history (Pang et al., 2019). These cases are thought to result from a combination of environmental factors and complex gene–environment interactions by mechanisms that are not yet comprehensively understood (Dunn et al., 2019). Epigenetic factors, such as DNAm, represent one such molecular mechanism that could mediate the genetic and environmental underpinnings of the etiology of PD.

## 3 Genetic and environmental underpinnings of Parkinson’s disease

### 3.1 Genetic predisposition to Parkinson’s disease and the role of *SNCA*

The first genetic studies of PD in the late 1990s identified hereditary mutations in the 4q21–q23 region and the *SNCA* gene linked to the disease in specific families (Polymeropoulos et al., 1996, 1997). However, further studies showed no associations between these mutations and PD, suggesting that the genetic etiology of PD was more complex than initially thought (Muñoz et al., 1997; Scott et al., 1997, 1999; Farrer et al., 1998). Eventually, variants in 28 chromosomal regions formerly known as the *PARK* loci were shown to be associated with PD. Of these, six were confirmed to cause monogenic PD: *SNCA*, *LRRK2*, *Parkin*, *PINK1*, *DJ-1*, and *ATP13A2* (Klein and Westenberger, 2012). Additional risk genes have been uncovered by genotyping and whole-exome sequencing, including *GBA* and *VPS35* (Lin and Farrer, 2014). However, *SNCA* mutations are of particular interest, as they often lead to autosomal dominant, early-onset disease that presents with dementia (Klein and Westenberger, 2012).

The *SNCA* gene encodes α-Syn, a protein localized to synaptic terminals with roles in vesicle transport and dopamine release (Villar-Piqué et al., 2016). *SNCA* overexpression or mutation results in production of abnormal α-Syn aggregates known as Lewy bodies. Excessive Lewy body accumulation is thought to be toxic to dopaminergic neurons and is a major hallmark of PD pathology. In addition, α-Syn inclusions typically contain a high proportion of the protein phosphorylated at serine 129, which influences the impact of α-Syn on gene expression and DNA damage, and could therefore be relevant to the pathogenesis of the disease (Zhou et al., 2011; Pinho et al., 2019).

#### 3.1.1 *SNCA* and familial Parkinson’s disease

Multiple forms of *SNCA* variation have been linked to familial and sporadic PD. Inherited *SNCA* duplications and triplications alter dosage and can lead to early-onset PD, with severity correlated to the degree of *SNCA* overexpression (Lesage and Brice, 2009; Klein and Westenberger, 2012). Rare inherited *SNCA* point mutations, including A53T, E46K, and A30P, are also associated with severe disease. The biochemical characteristics of these *SNCA* variants have been primarily studied in cell culture and animal models. *SNCA* multiplications are often replicated by overexpressing human α-Syn anywhere from two-to fivefold, producing excess protein, which is also seen in human multiplication carriers (Oliveira et al., 2015; Paiva et al., 2017; Wassouf et al., 2018). *SNCA* point mutations can also be studied by expressing mutant human α-Syn constructs in cell lines or animal models (Jo et al., 2002; Kontopoulos et al., 2006; Freichel et al., 2007; Paiva et al., 2017, 2018).

#### 3.1.2 *SNCA* and sporadic Parkinson’s disease

*SNCA* can influence susceptibility to not only familial PD, but also to sporadic PD. The contributions of *SNCA* and other genes to the sporadic form of the disease have primarily been assessed using linkage or genome-wide association studies (GWAS) in human populations. Minor variants in a large number of genes discovered in these populations, including *UCHL1*, *MAPT*, and *APOE*, have been shown to influence penetrance, age of onset, severity, and progression of PD (Lesage and Brice, 2009; Klein and Westenberger, 2012). The total contribution of common genetic variants to sporadic PD risk was estimated to be 22% (Nalls et al., 2019). GWAS have implicated up to 90 loci in sporadic PD, including several SNPs at different positions in *SNCA* (International Parkinson’s Disease Genomics Consortium and Wellcome Trust Case Control Consortium, 2011; Escott-Price et al., 2015; Nalls et al., 2014, 2019; Chang et al., 2017; Goldman et al., 2019; Blauwendraat et al., 2020; Ihle et al., 2020). Variations in the length of the REP1 allele, a dinucleotide repeat found in the *SNCA* promoter, have also been shown to influence PD susceptibility (Chiba-Falek and Nussbaum, 2001; Maraganore et al., 2006).

### 3.2 Environmental factors influencing Parkinson’s disease risk

Several lines of evidence from human and animal studies indicate that lifestyle-related factors are associated with PD risk. In human epidemiological studies, smoking, coffee consumption, and exercise have been shown to reduce the risk of developing PD, while pesticide exposure, dairy consumption, and brain injury have been shown to increase risk of PD (Ascherio and Schwarzschild, 2016). The influence of the environment on PD risk may be partially mediated through alterations to the epigenome (Ho et al., 2012; Angelopoulou et al., 2022). This review focuses on the epigenomic impacts of pesticide exposure, one of the best-validated risk factors for PD, and physical activity, one of the best-validated protective factors against PD.

#### 3.2.1 Pesticide exposure

The influence of pesticide exposure on PD risk was first discovered in observational studies (Supplementary Table S3). In 1983, 1-methyl-4-phenyl-1,2,3,6-tetrahydropyridine (MPTP), a compound found in heroin that has properties similar to the herbicide paraquat, was found to cause parkinsonism in drug users (Langston et al., 1983; Pang et al., 2019). Elevated PD incidence rates have also been reported in communities with pesticide-contaminated well water as the main source of drinking water (Rajput et al., 1987; Logroscino, 2005). Subsequently, epidemiological studies linked pesticide exposure to increased risk of sporadic PD in larger populations (Elbaz et al., 2009; Tanner et al., 2011; Pouchieu et al., 2018) (Supplementary Table S3). These pesticides and other environmental neurotoxins have been suggested to enter the body through the olfactory system or digestive tract, inducing inflammation, oxidative stress, and/or mitochondrial toxicity, and may initiate α-Syn neuropathology (Tanner et al., 2011; Chen and Ritz, 2018). Exposure to these chemicals was shown to induce parkinsonian molecular and behavioral phenotypes in cell culture and animal models, lending experimental support to studies performed in human populations (Marey-Semper et al., 1995; Betarbet et al., 2000; Przedborski et al., 2001; McCormack et al., 2002; Peng et al., 2004) (Supplementary Table S3).

Several lines of evidence also suggest that pesticide exposure interacts with the genome and epigenome to influence PD risk (Supplementary Table S4). For example, the *LRRK2* G2019S mutation increases the inflammatory response to paraquat in mice; *DAT* variants and herbicide exposure can jointly influence PD risk; and *CYP2D6* variants are associated with altered DNAm and PD risk (Elbaz et al., 2004; Ritz et al., 2009; Tiili et al., 2015; Rudyk et al., 2019). Taken together, these observations suggest that pesticides may impact PD etiology through direct and/or indirect mechanisms. Further research regarding the interactions of such exposures with the genome and epigenome are required to identify specific pathways that lead to PD and to facilitate the targeting of these pathways in personalized medicine strategies.

#### 3.2.2 Physical activity and enriched environment

In addition to the impact of toxin exposures on PD risk, the role of lifestyle-related factors in PD prevention has also been investigated. Exercise has been highlighted as having a protective effect against neurodegeneration, and was shown to be associated with reduced risk of PD in prospective cohort studies (Sasco et al., 1992; Chen et al., 2005; Crotty and Schwarzschild, 2020) (Supplementary Table S5). In addition, randomized controlled trials suggested that exercise may also improve motor symptoms of PD (Fisher et al., 2008; Rawson et al., 2019; Sujkowski et al., 2022). Exercise has been shown to ameliorate the effects of aging and neurodegeneration by a number of mechanisms, including increases in brain-derived neurotrophic factor (BDNF) and dopamine release, increased synaptic plasticity, and stabilization of antioxidant responses (Wassouf and Schulze-Hentrich, 2019; Crotty and Schwarzschild, 2020). In rodents, the neuroprotective effects of exercise, increased cognitive stimulation, and increased social stimulation can be modeled with an “enriched environment” (EE) paradigm, consisting of housing more mice per cage with increased access to toys and exercise wheels (Wassouf and Schulze-Hentrich, 2019). Housing mice in an EE has been shown to reduce aging and inflammatory phenotypes, and to remodel gene expression, DNA modifications, and histone modifications (Irier et al., 2014; Wassouf et al., 2018; Zhang et al., 2018; Espeso-Gil et al., 2021; Zocher et al., 2021) (Supplementary Table S5). Exercise is also associated with reduced α-Syn levels in the mouse brain (Zhou et al., 2017). Taken together, the results of human and rodent studies provide good support for the neuroprotective effects of exercise against PD and suggest several potential underlying mechanisms. Future studies should continue to build upon this work by assessing the interactions of exercise with PD genetic risk, environmental exposures, and the epigenome.

## 4 Epigenetic embedding of genetic and environmental influences

### 4.1 Epigenetics, DNA methylation, and DNA hydroxymethylation

Waddington introduced the concept of the “epigenetic landscape” in 1957, referring to the effects of gene regulation on phenotype during cellular differentiation and development (Waddington, 1957). Riggs later defined “epigenetics” as mitotically heritable factors other than the DNA sequence that can shape cellular phenotype (Russo et al., 1996). A number of adjustments to this definition have since been proposed, which take into account chromosomal structure, cellular reprogramming, and cellular tissue composition (Bird, 2007; Lappalainen and Greally, 2017).

Modern definitions of “epigenetics” typically encompass protein and DNA modifications that can affect transcription, such as DNAm and histone posttranslational modifications. Epigenetic factors play crucial roles in cellular/tissue differentiation, development, and aging. For example, DNAm helps to guide neurogenesis, and widespread DNAhm changes are observed at genes involved in synaptic function, dendrite morphogenesis, and axon guidance in the developing fetal brain (Spiers et al., 2017; Stricker and Götz, 2018). Aging is also associated with widespread changes to DNAm at genes involved in DNA repair, apoptosis, cellular metabolism, and other key pathways (Jones et al., 2015; Slieker et al., 2016; Horvath and Raj, 2018).

#### 4.1.1 CpG methylation

**FIGURE 1:**
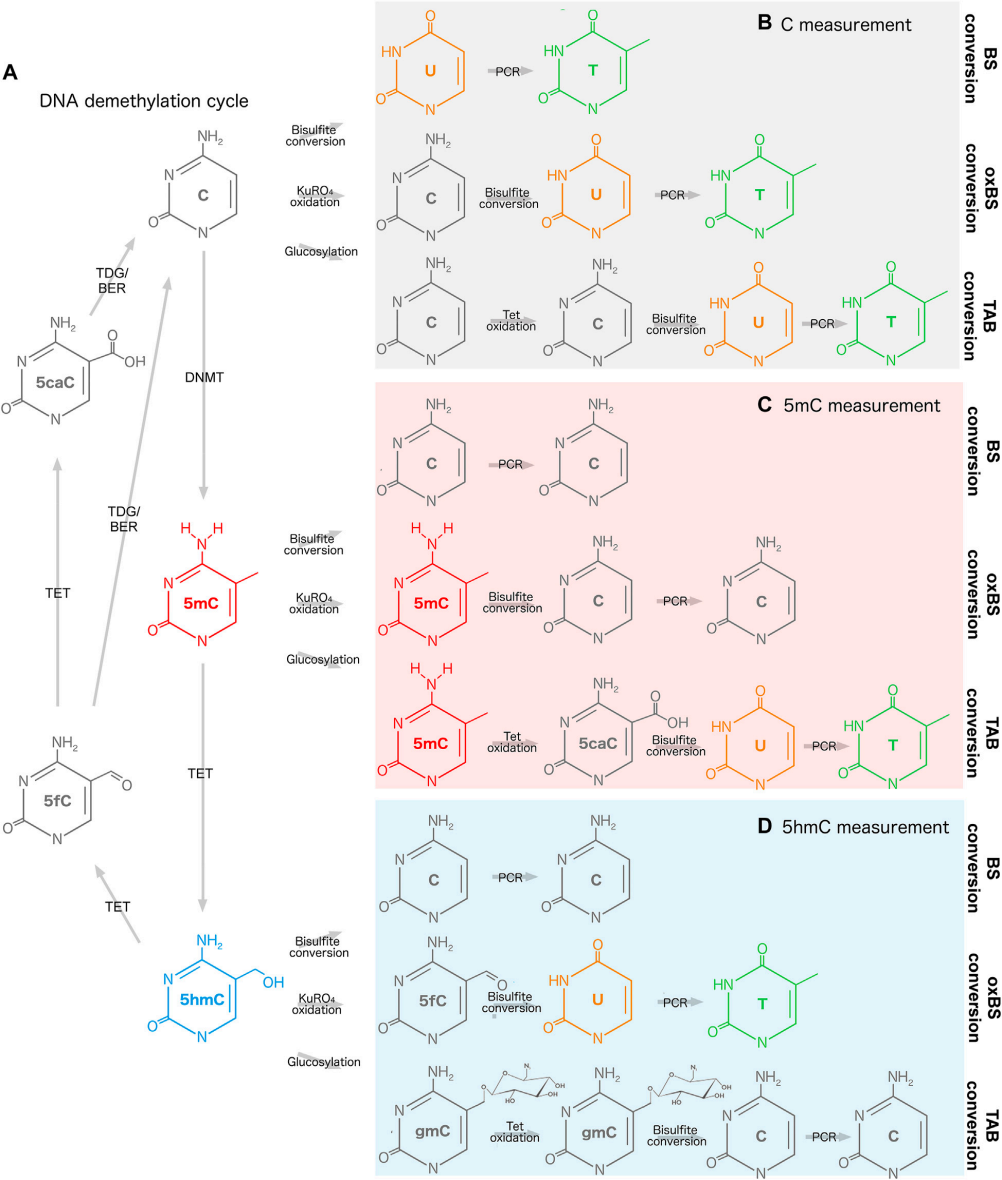
Measurement of DNA methylation and hydroxymethylation. **(A)** DNA demethylation cycle and reaction intermediates. **(B)** Bisulfite, oxidative bisulfite, and TET-assisted bisulfite conversion of cytosine. **(C)** Bisulfite, oxidative bisulfite, and TET-assisted bisulfite conversion of 5mC. **(D)** Bisulfite, oxidative bisulfite, and TET-assisted bisulfite conversion of 5hmC. 5caC, 5-carboxylcytosine; 5fC, 5-fluorocytosine; 5hmC, 5-hydroxymethylcytosine; 5mC, 5-methylcytosine; BER, base excision repair; BS, bisulfite; C, cytosine; DNMT, DNA methyltransferase; gmC, β-glucosyl-5-hydroxymethylcytosine; oxBS, oxidative bisulfite; PCR, polymerase chain reaction; T, thymine; TAB, TET-assisted bisulfite; TDG, thymine DNA glycosylase; TET, Ten-Eleven Translocation; U, uracil.

DNA methylation is one of the most extensively characterized epigenetic marks in human population studies, and is involved in all of the above processes from differentiation to development, aging, and disease (Cutfield et al., 2007; Levine et al., 2018; Stricker and Götz, 2018). Both cytosine and adenine bases can be methylated, with 5-methylcytosine (5mC) representing the most commonly methylated DNA base in the human genome (Kumar et al., 2018). 5mC has a methyl group attached to the fifth carbon of the pyrimidine ring of cytosine and can occur in the CpG, CHG, or CHH context (with CHG and CHH being examples of non-CpG methylation, referred to as CpH methylation where H represents either A, C, or T). Approximately 4% of the cytosines in the human genome are methylated, including 80% of CpG dinucleotides and 2%–6% of CpH sites (Lister et al., 2013; Kumar et al., 2018). Cytosine methylation is catalyzed by the DNA methyltransferase (DNMT) family of enzymes, including DNMT1, which maintains DNAm patterns during cell division, and DNMT3A and DNMT3B, which deposit *de novo* DNAm (Kumar et al., 2018) (Figure 1A).

CpG sites are found in varying densities across the genome, with the highest concentration in evolutionarily conserved CpG island promoters. DNAm at regions flanking CpG islands, referred to as CpG shores, plays an important role in establishing differences between tissues during development (Irizarry et al., 2009; Lokk et al., 2014). The roles of CpG methylation in other areas of the genome are complex and highly dependent on context, varying according to tissue, CpG density, and other factors, including developmental stage and age. Deposition of DNAm at CpG island promoters can silence gene expression through steric hindrance of transcription initiation complex binding or recruitment of additional repressive protein complexes through methyl-binding domains (Deaton and Bird, 2011). Conversely, CpG methylation in gene bodies is often associated with active transcription and may play a role in splicing regulation (Jones, 2012). Finally, DNAm may be laid down at some promoters and enhancers following gene silencing to maintain a transcriptionally repressive state (Jones, 2012).

#### 4.1.2 CpG hydroxymethylation

The Ten-Eleven Translocation (TET) family of methylcytosine dioxygenases actively removes DNAm from CpG sites, producing 5-hydroxymethylcytosine (5hmC) as the first intermediate in a multistep oxidation process (Wu and Zhang, 2011) (Figure 1A). While DNAhm was initially hypothesized to function only as a reaction intermediate, further studies supported an independent function for DNAhm, particularly in the brain (Szulwach et al., 2011; Wen and Tang, 2014; Kinde et al., 2015). DNAhm is highly enriched in human brain tissue, accounting for 17%–30% of all modified cytosines in the adult frontal cortex (Lister et al., 2013; Wen et al., 2014). DNAhm patterns change in response to neuronal activity, development, and aging, and may be key components of normal brain function (Szulwach et al., 2011). This epigenetic mark is also found at lower levels across a variety of human tissues, and may be involved in regulation of tissue-specific gene expression (He et al., 2021). However, the role of 5hmC in human gene regulation has not been fully elucidated, and its relevance to neurological health and disease is currently an area of active research.

#### 4.1.3 Non-CpG methylation

In contrast to CpG methylation, CpH methylation occurs primarily in embryonic stem (ES) cells and neurons, and is estimated to represent half of the methylated cytosines in adult human and mouse neurons (Kinde et al., 2015). CpH methylation has been suggested to be a transcriptionally repressive mark due to its depletion in upstream, downstream, and body regions of actively transcribed genes (Lister et al., 2013; Guo et al., 2014; Kinde et al., 2015). The abundance of such CpH methylation in the brain and its alterations in disease suggest that this is an important epigenetic mark in the context of brain health and development (Blanch et al., 2016; Fuso et al., 2016; Nicolia et al., 2017). However, further research is needed to determine whether and to what degree CpH methylation is sensitive to environmental and lifestyle-related factors.

### 4.2 Factors influencing DNA methylation and DNA hydroxymethylation

DNA methylation can be influenced by a myriad of genetic and environmental factors, from SNPs and copy number variation (CNV) to exposures and lifestyle-related factors, such as diet, pollution, and stress. The impacts of these factors on CpG methylation have been well characterized, while their impacts on DNAhm and CpH methylation are less well understood. This section first addresses the influences of genetics and environment on CpG methylation patterns.

#### 4.2.1 Influences of genetics and environment on CpG methylation

##### 4.2.1.1 Methylation quantitative trait loci

Although DNAm has frequently been proposed as a mediator of environmental and lifestyle impacts on health and disease susceptibility, it has recently become clear that genetic variants have a significant influence on CpG methylation, in some cases to a larger degree than environmental exposures. Genetic influences account for approximately 20%–80% of overall variation in DNAm, with a mean genome-wide CpG methylation heritability of 0.19 in twins (Gertz et al., 2011; Cheung et al., 2017; Husquin et al., 2018; Villicaña and Bell, 2021). Genetically driven CpG methylation is often considered in the context of methylation quantitative trait loci (mQTLs), which refer to SNPs where genotype impacts DNAm level (Banovich et al., 2014). It has been reported that 93% of mQTLs influence CpG methylation in cis, within 1 Mb of the affected CpG site (Gaunt et al., 2016). Approximately half of all mQTLs are located in introns, and they are also more likely to be represented in microRNA binding sites than expected by chance (Smith et al., 2014). Many mQTLs discovered in blood are consistent across tissues, developmental stages, and ethnicities. For example, two studies found that 18.5%–31.6% of blood mQTLs were also present in brain; 34%–73% of blood mQTLs overlapped between blood, brain, and saliva; 44.1%–50.7% overlapped between umbilical cord blood and adult peripheral blood; and 21.3%–69.5% of umbilical cord blood mQTLs overlapped between African and Caucasian infants (Smith et al., 2014; Liu et al., 2019). mQTL status is also highly consistent throughout life from birth to middle age (Gaunt et al., 2016).

Identification of relevant mQTLs can aid in understanding the mechanisms underlying complex diseases with varied genetic and environmental etiologies. For example, loci shown in GWAS to be associated with neurological and psychiatric disorders, cholesterol level, bone density, and blood pressure are enriched in mQTLs, suggesting that genetically driven CpG methylation patterns may be important in the context of health and disease (Gaunt et al., 2016; Liu et al., 2019). Several PD-associated SNPs have been reported or predicted to affect DNAm based on publicly available datasets, and PD GWAS data have been used to predict DNAm levels at thousands of CpGs, suggesting a key role of genetic variation in determining PD-associated DNAm patterns (International Parkinson’s Disease Genomics Consortium and Wellcome Trust Case Control Consortium, 2011; Nalls et al., 2014; Rawlik et al., 2016; Kia et al., 2021). It will be necessary to determine the degrees to which genetic variants affect CpG methylation as well as the directions of these effects to understand complex disorders, and it may be possible to apply such approaches to peripheral tissues to obtain informative results (Islam et al., 2019).

##### 4.2.1.2 Other genetic influences on CpG methylation

It is also important to consider the influences of non-SNP genetic factors on CpG methylation in the context of gene regulation, health, and disease. For example, allele-specific DNAm occurs at imprinted regions and as a consequence of X chromosome inactivation, a phenomenon in which one of the X chromosomes in females is epigenetically silenced; defects in these mechanisms can lead to developmental disorders (Horsthemke, 2014; Wang H. et al., 2019a). Deletions, duplications, or rearrangements of gene regions, entire genes, or larger genomic segments, such as CNV, can also affect CpG methylation through direct or indirect mechanisms. Some CNV regions may physically interact with CpG sequences, and disruption of these interactions affects the ability of DNMTs to access these sequences and catalyze CpG methylation. Other CNV contain transcription factor binding sites, which may alter gene expression and transcription-associated DNAm changes. Alternatively, dosage changes in cell signaling proteins and messengers can affect physiological signaling cascades that ultimately influence gene transcription and DNAm (Shi et al., 2020). In the context of familial PD, whole-gene multiplications of the *SNCA* locus may impact the epigenome through some of these mechanisms. For example, increased dosage of α-Syn in dopaminergic neurons impacts genome-wide DNAm and DNAhm patterns (Schaffner et al., 2022). However, the molecular epigenetic effects of *SNCA* CNV in human PD patients have not been fully elucidated.

##### 4.2.1.3 Environmental influences on CpG methylation

While genetic factors are known to influence DNAm patterns, there is also a large body of evidence for the plasticity of CpG methylation in response to the environment (Feinberg, 2007; Aguilera et al., 2010; Ho et al., 2012; Cavalli and Heard, 2019; Angelopoulou et al., 2022). A number of factors, including smoking, nutrition, and early life stress, are associated with altered DNAm in humans (Lim and Song, 2012; Lee and Pausova, 2013; Matosin et al., 2017). These environmental and lifestyle-related factors also influence PD risk, which may be partially mediated by impacts on the epigenome (Chen and Ritz, 2018; Angelopoulou et al., 2022). Therefore, it is crucial to understand the environmental influences on CpG methylation to investigate the role of DNAm in PD etiology, for the design of epigenetic studies of PD, for selecting appropriate statistical approaches to minimize confounding, and to explore whether lifestyle interventions for PD act through epigenetic mechanisms.

Environment-induced DNAm changes can also be partly influenced by genetic background, which will be a key consideration in the design of future epigenetic studies of PD. For example, in neonatal umbilical cord blood, the interaction of genotype and *in utero* environment was found to explain 75% of DNAm variation across variably methylated regions (Teh et al., 2014). A similar analysis in neonates showed that additive and interaction effects of genotype and environment explained 29.41% and 40.58% of DNAm variance, respectively, while environment alone explained only 0.03% (Czamara et al., 2019). Environment-driven variation in the epigenome has been proposed to act as a “second hit” on top of genotype, possibly triggering onset of disorders to which an individual is genetically predisposed, such as CNV-associated neuropsychiatric conditions (Girirajan et al., 2010). The influence of environmental and lifestyle-related factors on DNAm may also partially ameliorate disease risk. For example, exercise is associated with DNAm changes in skeletal muscle, and bariatric surgery has been shown to partially reverse diet-induced DNAm changes in glucose uptake genes (Barrès et al., 2012; Multhaup et al., 2015). The impact of environment on the DNA methylome is particularly crucial during critical periods of early development, and DNAm has been proposed to be one of the mediators of developmental origins of health and disease (Barker, 1990; Cutfield et al., 2007). There is currently a great deal of active research regarding the possibility of disease prevention strategies targeting the epigenome, as well as epigenetic therapies that could be applied later in life.

#### 4.2.2 Influence of genetics and environment on CpG hydroxymethylation

Similar to CpG methylation, it is also important to understand the impacts of genetics and environment on CpG hydroxymethylation and non-CpG methylation for epigenetics studies of PD. As these two marks are enriched in the brain, have the potential to influence gene transcription, and may also be affected by PD genetic and environmental risk factors, it may be prudent to incorporate analysis of DNAhm and/or CpH methylation into studies of PD etiology.

The degree to which DNAhm is susceptible to genetic and environmental influence is still under investigation. However, a small number of hydroxymethylation quantitative trait loci (hmQTLs) have been identified in the fetal brain, and human tissue-specific differentially hydroxymethylated regions have been shown to contain SNPs linked to tissue-specific diseases and phenotypes (Spiers et al., 2017; He et al., 2021). Changes in 5hmC have also been reported to be associated with exposure to stress, environmental enrichment, toxins, and diet (Irier et al., 2014; Hack et al., 2016; Kochmanski and Bernstein, 2020). Finally, several studies have implicated DNAhm in neuronal activity-regulated gene expression, learning and memory, and neuroplasticity, all of which are likely to involve external environmental changes and experience (Kaas et al., 2013; Li et al., 2014). However, some of these studies measured 5hmC on a global level. A shift toward base-pair-resolution 5hmC analysis is needed to develop a comprehensive understanding of the genes and pathways affected by environmental alterations in this epigenetic mark and its significance for disease development and prevention (Kochmanski and Bernstein, 2020).

#### 4.2.3 Influence of genetics and environment on non-CpG methylation

Little is known about the impacts of genetics and environment on CpH methylation patterns. Some allele-specific CpH methylation has been observed in the context of schizophrenia, with lower levels of CpH methylation surrounding SNPs that correspond to schizophrenia risk haplotypes (Alfimova et al., 2020). CpH methylation changes have also been reported at 750 cytosines, primarily occurring in introns, in the hippocampi of mice housed in EE (Zocher et al., 2021). While DNAm at introns has the potential to influence transcription, differential CpH methylation was also shown to be depleted at promoters, CpG islands, and CpG shores in this study, making it difficult to draw any definitive conclusions about potential gene regulatory impacts (Zocher et al., 2021). Neuronal CpH methylation has been suggested to be more sensitive to environmental stimuli than neuronal CpG methylation due to its sparsity and replication independence (Fuso and Lucarelli, 2019). Further research is needed to determine whether this holds true, and to elucidate the full extent of genetic and environmental effects on CpH methylation.

### 4.3 Methods for measuring DNA modifications

Both when evaluating existing PD EWAS and when designing new experiments, it is valuable to consider the method used to profile genome-wide DNAm to understand its limits and biases. For example, studies in brain tissue may be biased by high levels of DNAhm, which are picked up in one compound signal when bisulfite conversion of DNA is applied (Darst et al., 2010). If bisulfite conversion is paired with oxidative or TET-assisted bisulfite conversion, 5mC and 5hmC levels can be estimated simultaneously (Booth et al., 2013; Yu et al., 2018) (Figure 1). The platform used for DNAm profiling will also influence the results. Illumina microarrays are often used for population-level studies and provide good coverage of regulatory elements, but overall sparse coverage of the genome (Bibikova et al., 2009; Pidsley et al., 2016). In contrast, next-generation sequencing approaches are often used with fewer samples, but may cover the genome at greater depth (Gu et al., 2011; Kernaleguen et al., 2018). There are also a multitude of statistical considerations for quality control and normalization of microarray and sequencing data, simultaneous 5mC/5hmC estimation in paired samples, and deconvolution of cell types in heterogeneous tissues (Krueger and Andrews, 2012; Guintivano et al., 2013; Pidsley et al., 2013; Liu and Siegmund, 2016; Lunnon et al., 2016; Xu et al., 2016; Kaushal et al., 2017; Zhang Y. et al., 2022a). Robust DNA modification studies should take all of the above into account to reduce bias and increase inferential capability. Further considerations for DNA modification profiling are reviewed in Laird, 2010 and Skvortsova et al., 2017.

## 5 Epigenetic contribution to Parkinson’s disease

**FIGURE 2:**
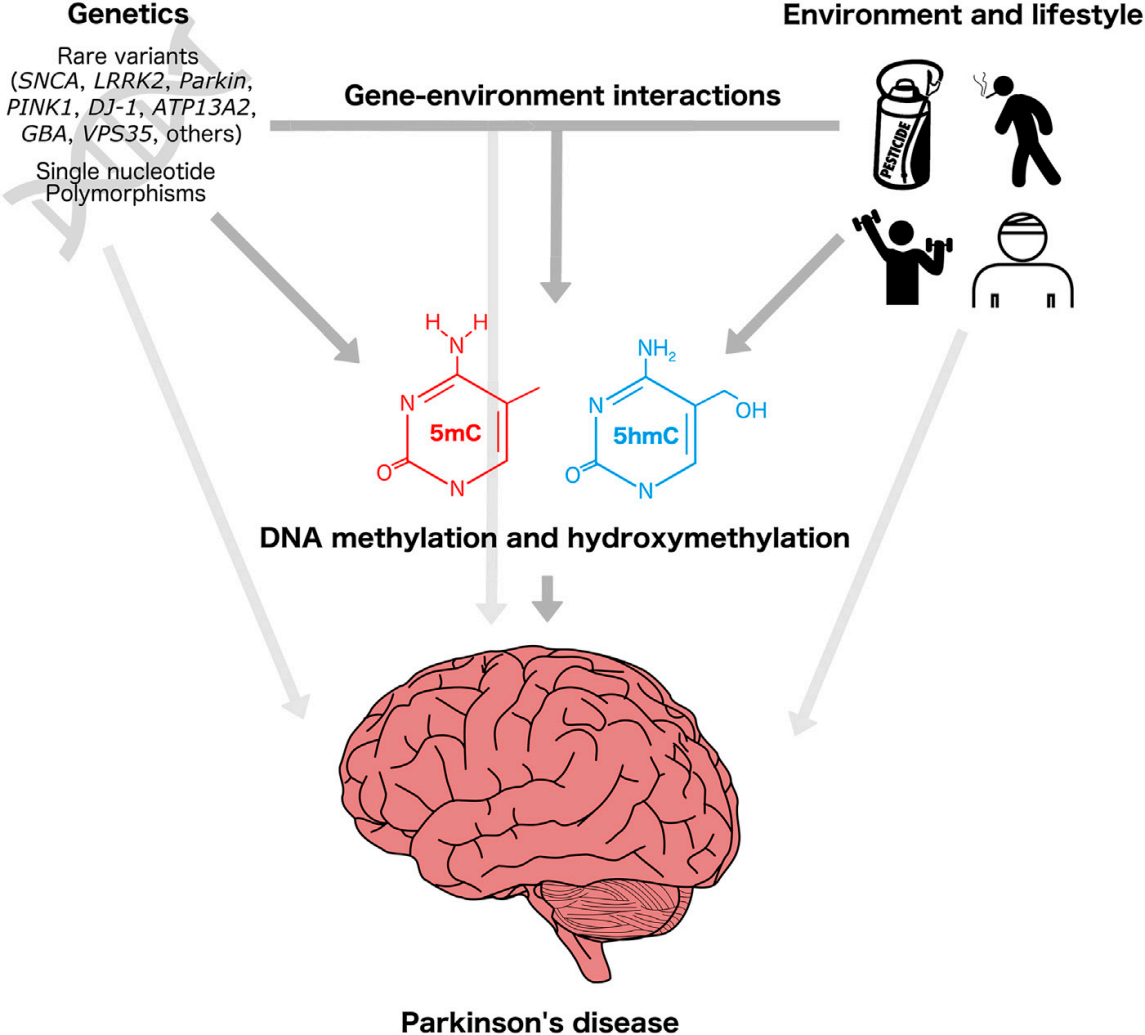
Genetic, environmental, and epigenetic underpinnings of Parkinson’s disease. Individual genetic background, environmental exposures (e.g., pesticides), and lifestyle-related factors (e.g., exercise, smoking, head trauma) influence PD risk. These factors may act directly or indirectly through modification of DNA methylation and/or DNA hydroxymethylation patterns, which in turn can influence the regulation of genes involved in neurodegenerative pathways. Head injury icon created by George E. Thomposon (thenounproject.com).

DNAm and DNAhm are particularly attractive as candidates to identify and characterize gene–environment interactions in PD because of the large proportion of sporadic cases, and as potential modifiers of familial disease (Figure 2). Local and genome-wide DNAm alterations and impacts on the DNAm machinery have been reported to be associated with genetic and environmental risk factors for PD, suggesting that DNAm and/or DNAhm could be involved in the etiology of the disease (Dunn et al., 2019; Angelopoulou et al., 2022). Some of these DNAm changes are correlated with changes in gene expression, and may therefore represent functionally relevant molecular profiles involved in the development of PD. For example, decreased DNAm at *SNCA* intron one is associated with increased *SNCA* mRNA expression in PD patients (Jowaed et al., 2010). Differentially methylated genes in PD patients are enriched for cell–cell communication and apoptotic pathways, also suggestive of a potential etiological role of this epigenetic modification (Kaut et al., 2012; Masliah et al., 2013).

In addition to its usefulness in understanding the etiology of PD, DNAm may represent a suitable early-stage biomarker for the disease. DNAm has characteristics that make it an ideal biomarker due to its relative stability and detectability in peripheral tissues, such as blood and saliva (Edgar et al., 2017; Islam et al., 2019). One study in matched brain and blood samples showed that CpG methylation patterns associated with PD were approximately 30% concordant across these tissues, suggesting that certain loci may be able to act as biomarkers in blood (Masliah et al., 2013).

### 5.1 Influence of *SNCA* and other Parkinson’s disease risk genes on DNA methylation

**FIGURE 3:**
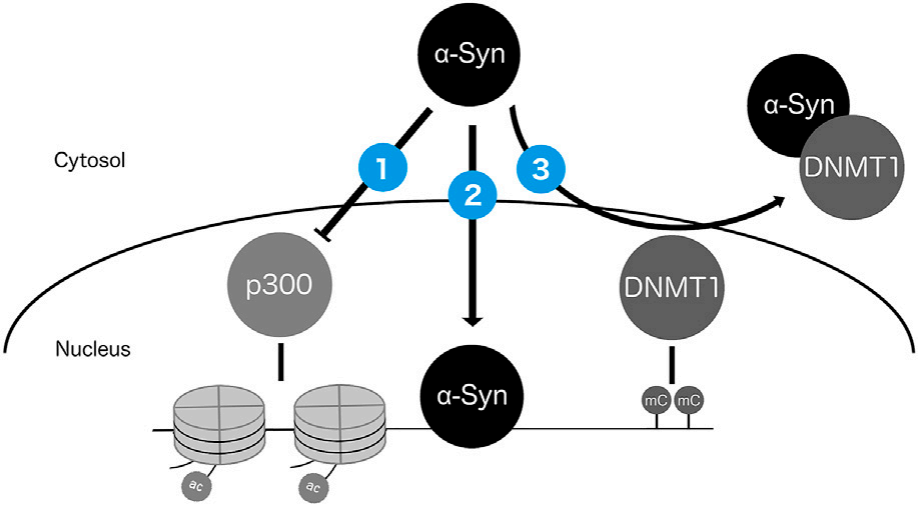
Potential mechanisms by which nuclear α-Syn protein can impact the epigenome. α-Syn can (1) inhibit activity of histone acetyltransferase p300 (Kontopoulos et al., 2006; Jin et al., 2011; Paiva et al., 2017); (2) directly bind DNA (Martins et al., 2011; Davidi et al., 2020); and (3) sequester DNMT1 from the nucleus to the cytosol (Desplats et al., 2011).

Studies using animal and cell culture models showed that the protein product of the PD risk gene *SNCA*, α-Syn, is localized to the nucleus and has several potential avenues for impacting epigenetic regulation (Figure 3). First, nuclear α-Syn can reduce p300 histone acetyltransferase activity and inhibit histone H3 acetylation (Kontopoulos et al., 2006; Jin et al., 2011; Paiva et al., 2017). Second, α-Syn can bind to DNA, regulating the transcription of genes responsive to retinoic acid signaling and relevant for PD (Martins et al., 2011; Davidi et al., 2020). Third, α-Syn was shown to sequester the maintenance DNA methylation enzyme DNA methyltransferase 1 (Dnmt1) from the nucleus to the cytoplasm in mice, potentially reducing the capacity for maintenance of CpG methylation (Desplats et al., 2011). In addition to these mechanisms, the presence of excess wild type or mutant α-Syn protein throughout the cell may impact epigenetic modifications indirectly as a consequence of altered signaling cascades resulting in epigenetic and transcriptional alterations, changes to cellular metabolism, and/or DNA damage resulting from α-Syn toxicity (Emamzadeh, 2016; Paiva et al., 2017). While these studies provided strong evidence that α-Syn can alter the epigenome, the impacts of expressing α-Syn and its variants on the DNAm level have not been fully characterized. Some experiments have addressed the locus-specific CpG methylation status of mutant α-Syn in lymphoblastoid cells, or investigated the epigenome-wide impacts of other PD-associated mutations, such as *LRRK2* G2019S, in PD patient-derived induced pluripotent stem cells (iPSCs) (Voutsinas et al., 2010; Fernández-Santiago et al., 2015, 2019). Recently, wild-type and A30P mutant α-Syn were shown to alter CpG methylation at thousands of loci in dopaminergic neurons, with correlations to expression of glutamate signaling genes (Schaffner et al., 2022). Studies such as these to characterize the effects of α-Syn on the DNA methylome will increase our understanding of the mechanisms underlying genetically driven PD, and whether the epigenetic consequences of *SNCA* variants can be reversed by lifestyle interventions or targeted therapies, such as epigenome editing (Kantor et al., 2018).

To date, few population-level studies of sporadic PD have addressed the contribution of genetic variants to DNAm patterns. DNA methylation levels of PD-associated SNPs discovered by GWAS have been assessed, and other studies have used public datasets to associate SNP genotypes with DNAm levels of PD-associated CpGs (International Parkinson’s Disease Genomics Consortium and Wellcome Trust Case Control Consortium, 2011; Nalls et al., 2014; Vallerga et al., 2020; Kia et al., 2021). The incorporation of mQTL analysis with matched samples and/or REP1 genotyping into further human cohort studies may be informative, and help to explain why some associations of DNAm with PD can be replicated, while others cannot.

### 5.2 Influence of Parkinson’s disease-associated environmental and lifestyle-related factors on DNA methylation patterns

#### 5.2.1 Pesticide exposure and DNA methylation

**FIGURE 4:**
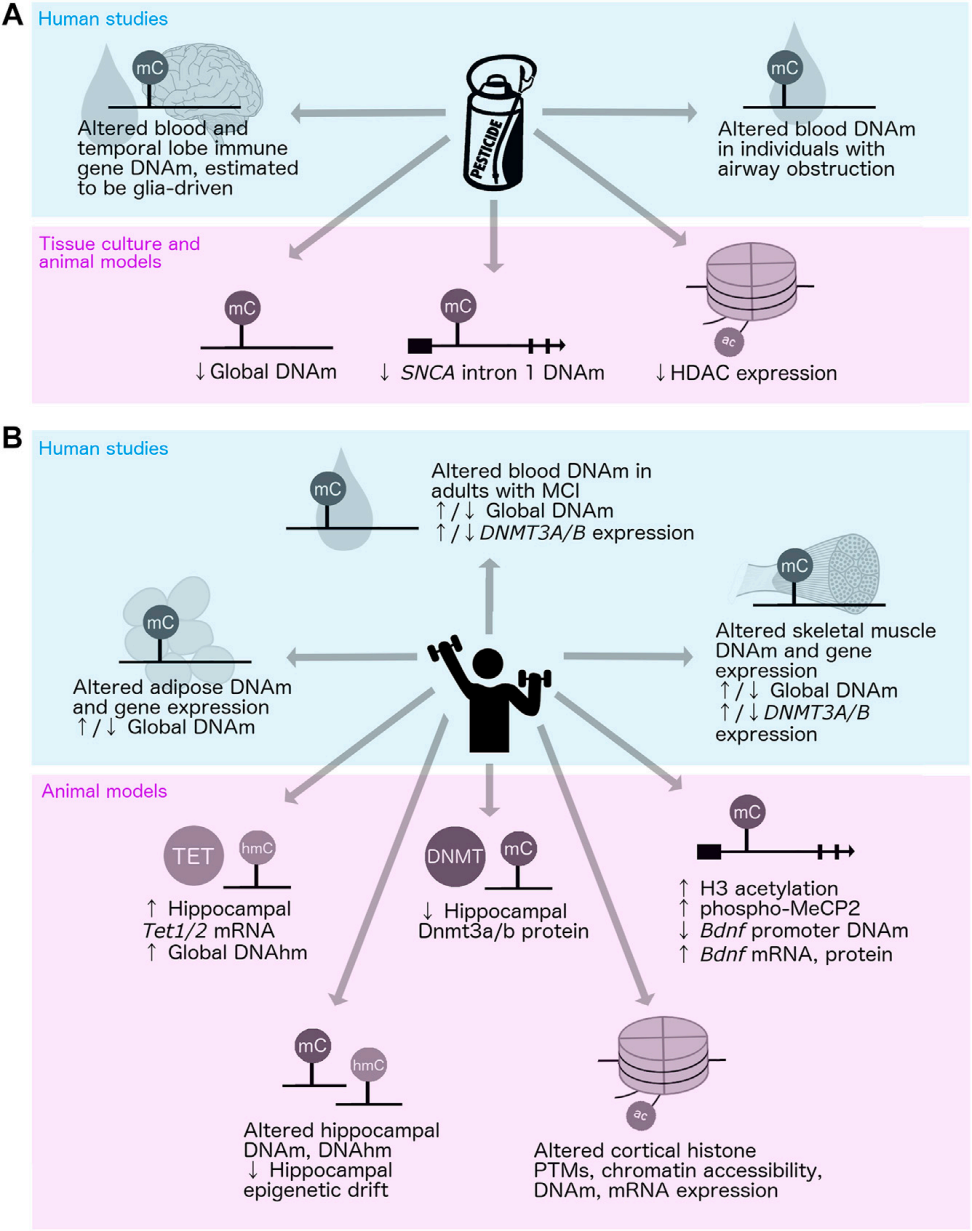
Impacts of pesticide exposure and physical activity on the epigenome. Up and down arrows (↑/↓) indicate conflicting results; i.e., both increases and decreases reported across different studies of the same mechanism. **(A)** Human (top: van der Plaat et al., 2018; Go et al., 2020; Furlong et al., 2020) and tissue culture/animal (bottom: Park et al., 2016; Wnuk et al., 2016; Zhao et al., 2019) studies of pesticide exposure and epigenetic alterations. **(B)** Human (top: Rönn et al., 2013; Lindholm et al., 2014; Światowy et al., 2021; Ngwa et al., 2022) and animal (bottom: Gomez-Pinilla et al., 2011; Elsner et al., 2013; Irier et al., 2014; Jessop and Toledo-Rodriguez, 2018; Wassouf et al., 2018; Zhang et al., 2018; Zocher et al., 2021; Espeso-Gil et al., 2021) studies of physical activity or enriched environment and epigenetic alterations. DNAhm, DNA hydroxymethylation; DNAm, DNA methylation; HDAC, histone deacetylase; MCI, mild cognitive impairment; PTMs, posttranslational modifications.

There have been only a few studies regarding the epigenetic impacts of PD-associated environmental and lifestyle-related factors, such as neurotoxin exposure and exercise. In the context of neurotoxins, one group investigated DNAm patterns in PD patients with and without pesticide exposure, and other studies have examined the impacts of pesticide exposure in healthy individuals; however, there have been no studies involving the assessment of PD patients and controls simultaneously (van der Plaat et al., 2018; Go et al., 2020) (Figure 4A). Global DNAm loss has been reported in organochlorine-exposed hippocampal cell culture, and site-specific DNAm changes at the first intron of *SNCA* were observed in an MPTP-induced mouse model of PD (Wnuk et al., 2016; Zhao et al., 2019) (Figure 4A). Pesticide exposure may also alter histone acetylation in PD, as MPTP-treated cells and mice showed reduced histone deacetylase (HDAC) expression (Park et al., 2016) (Figure 4A). This pathway may be a viable target for intervention, as a recent study showed that naturally occurring short-chain fatty acids produced by gut microbes influence HDAC activity and can protect against rotenone-induced toxicity in rat dopaminergic neurons (Zhang Z. et al., 2022b). Additional human population studies to assess the associations between pesticide exposure and DNAm in PD and experimental studies in model systems to investigate whether it is mechanistically involved in the pathogenesis of PD would help to further elucidate the potential role of the epigenome in mediating the effects of pesticide exposure on disease risk.

#### 5.2.2 Physical activity, enriched environment, and DNA methylation

The impacts of PD-associated behavioral and lifestyle-related factors on DNAm have also been assessed to some extent. The effects of physical activity on DNAm in humans have been widely studied, primarily in skeletal muscle, adipose tissue, and blood (Figure 4B). However, conflicting results have been reported regarding whether and in what direction exercise impacts global DNAm patterns, most likely due to inconsistencies in the age of subjects and the type and duration of exercise applied in these investigations (Światowy et al., 2021). Interestingly, 6 months of exercise was shown to alter DNAm at genes related to amyloid biology, protein trafficking, and lipoprotein regulation in the blood of adults with mild cognitive impairment, suggesting possible impacts on neurologically relevant gene regulation (Ngwa et al., 2022). Most of our knowledge regarding the impacts of exercise on the brain epigenome has come from studies in rodent models (Figure 4B). Physical activity has been shown to increase *Tet1* and *Tet2* mRNA expression and DNAhm levels in the mouse hippocampus, and to decrease Dnmt1 and Dnmt3b protein levels in the rat hippocampus (Elsner et al., 2013; Jessop and Toledo-Rodriguez, 2018). Exercise was also shown to elevate the levels of phosphorylated MeCP2 in the rat hippocampus, which is associated with reduced DNAm at the *Bdnf* promoter and increased levels of *Bdnf* mRNA and protein expression (Gomez-Pinilla et al., 2011). These studies point to DNAm as one mechanism mediating BDNF upregulation and neuroprotection associated with physical activity.

The effects of exercise and increased cognitive and social stimulation on the epigenome have also been studied in rodents using EE paradigms (Figure 4B). These studies showed that environmental enrichment can alter hippocampal DNAm and DNAhm, and prevent some *SNCA*-induced alterations in gene expression (Irier et al., 2014; Wassouf et al., 2018; Zhang et al., 2018; Zocher et al., 2021). Intriguingly, EE was shown to reduce age-related epigenetic drift at the level of DNAm in the hippocampus, and to alter histone modifications, chromatin accessibility, DNAm, and gene expression in the cortex in mice (Espeso-Gil et al., 2021; Zocher et al., 2021). These studies support a potential role of DNAm as a mediator of the impacts of behavior and lifestyle-related factors on PD risk.

### 5.3 CpG methylation alterations in individuals with Parkinson’s disease

Global, gene-specific, and genome-wide DNAm alterations have been identified in PD patients. Lower levels of global DNAm have been reported in the brains of PD patients compared to controls (Kaut et al., 2012). This may be related to α-Syn-mediated sequestration of DNMT1 from the nucleus to the cytoplasm and/or targeting of DNMT1 by microRNA(s), perturbing neuronal DNAm patterns (Desplats et al., 2011; Zhang et al., 2021). Alterations to DNAm have also been demonstrated at specific loci in PD across several tissues, including the postmortem brain, PD patient-derived neurons, blood, and saliva (Supplementary Tables S1, S2). Increased DNAm has been reported at intron one of the *SNCA* gene in PD, but a correlation with α-Syn expression is controversial (Jowaed et al., 2010; de Boni et al., 2015). In addition to *SNCA*, several EWAS have reported site-specific changes in CpG methylation across a number of genes in individuals with PD, including *CYP2E1*, *MIR886*, *MAPT*, *LARS2*, and *SLC7A11* (Masliah et al., 2013; Rawlik et al., 2016; Chuang et al., 2017; Henderson-Smith et al., 2019; Vallerga et al., 2020; Kaut et al., 2022) (Supplementary Tables S1, S2). Attempts to replicate the results of these EWAS have often failed due to factors such as cell type heterogeneity, small sample sizes, and differences in study design and data collection/processing. Larger populations and more robust methods of accounting for tissue differences, disease heterogeneity, and mixed ethnicity of study populations are required to produce reliable and replicable EWAS results for PD.

Aside from such studies focusing on DNAm patterns associated with PD diagnosis, some groups have begun to investigate how medications and pesticide exposures can affect DNAm patterns in PD (Schmitt et al., 2015; Furlong et al., 2020). However, it is unclear whether environmental and lifestyle-related factors, such as pesticide exposure, coffee consumption, and diet, can influence or mediate DNAm changes associated with PD risk in undiagnosed individuals (Angelopoulou et al., 2022).

### 5.4 CpG hydroxymethylation alterations in individuals with Parkinson’s disease

The study of DNAhm patterns in PD is also in its infancy. Recent work has shown that 5hmC is enriched globally in the white matter of PD patients as well as at enhancers in the neurons of PD patients (Kaut et al., 2019; Marshall et al., 2020). One of these groups also determined that PD patients exhibit upregulation of TET2, and that TET2 depletion can prevent dopaminergic neuron loss in mouse and cell culture models of PD (Marshall et al., 2020). These observations represent promising early evidence that DNAhm may be involved in the pathogenesis of PD, and therefore DNAhm changes in PD warrant further characterization.

### 5.5 Non-CpG methylation in individuals with Parkinson’s disease

CpH methylation may also play a role in neurodegeneration, and has been shown to be enriched at genes involved in immune, neuroinflammation, and neurodegeneration-related pathways (Lee et al., 2020). CpH methylation changes have been reported in mitochondrial DNA from the substantia nigra of PD patients (Blanch et al., 2016). However, the majority of research in the context of PD disease etiology and biomarker potential to date has focused on CpG methylation. Future studies should continue to assess disease-associated cytosine modifications, including CpH methylation and DNAhm, to determine whether they have causal relationships with PD phenotypes.

## 6 Multi-omics integration approaches in neuroepigenetics research

Although both DNAm and DNAhm are interesting facets of the epigenome that may explain some of the variation observed in PD, the relationships of DNAm and DNAhm with gene expression are highly complex, making it difficult to draw functional conclusions from studies of the DNA methylome alone. Multi-omics profiling and data integration approaches can help to prioritize functionally relevant epigenetic loci, develop better predictive biomarkers, and in certain cases aid in determining causality (e.g., when genetic data are included and/or when data are sampled across multiple time points). This is particularly useful for understanding the etiology and progression of complex diseases and for developing personalized medicine strategies (Peterlin and Maver, 2012; Cazaly et al., 2019; Li et al., 2021).

Multi-omics integration can be accomplished using a wide range of approaches that vary in terms of their data inputs, assumptions, and qualitative versus quantitative nature, with no universally accepted “gold standard” (Richardson et al., 2016; Cazaly et al., 2019; Li et al., 2021). On the quantitative end, linear regression can be used to model one data type as the dependent variable and another as the independent variable (Stingo et al., 2011; Kim and Xing, 2012; Marttinen et al., 2014; Zhang et al., 2019). However, when both datasets are high dimensional, such as genome-wide DNAm, DNAhm, and gene expression profiles, it may be necessary to run thousands of models in a time- and computationally intensive manner, and multiple test correction can heavily penalize the results. Hierarchical clustering can also be used to find related features among such datasets (Curtis et al., 2012; Lock and Dunson, 2013; Singh et al., 2019). More qualitative approaches seek to identify common pathways, functions, and/or networks of genes profiled by multi-omics approaches, including gene ontology (GO) enrichment analysis, chromatin state prediction, and coexpression network analysis, among others (Ernst and Kellis, 2012; Wijetunga et al., 2017; Reimand et al., 2019). Finally, machine learning approaches can be applied to multiple omics layers in a supervised or unsupervised manner, selecting features that correlate with phenotype (Singh et al., 2019; Li et al., 2021).

While many different omics approaches have been applied in PD, only a few studies to date have combined multiple data types (Redenšek et al., 2018; Wang C. et al., 2019b; Henderson et al., 2021; Kia et al., 2021; Caldi Gomes et al., 2022). One group integrated genomics, transcriptomics, and proteomics using a position-based method, and discovered 29 dynamically regulated genes associated with PD (Maver and Peterlin, 2011). Another group compared the results of separate RNA-sequencing and proteomics analyses, and found that only 10/3,558 genes with differential mRNA expression in PD also had altered protein levels (Dumitriu et al., 2015). Importantly, this suggests that assumptions about downstream gene expression and function cannot be extrapolated from the results obtained using a single omics approach. More recently, two separate studies integrated gene expression and DNAm profiles in blood of PD patients. One study used linear regression to construct a biomarker panel of 85 genes with decreased expression and increased DNAm in PD, while the other applied a network-based approach and found six PD-related regulatory modules with altered expression and DNAm (Wang C. et al., 2019b; Henderson et al., 2021). A similar network approach was applied to discover modules of differentially expressed genes at the levels of mRNA, microRNA, and/or protein in PD (Caldi Gomes et al., 2022). Finally, multiple large-scale, publicly available omics datasets have been used to test hypotheses about how genetic variants influence gene expression, splicing, and DNAm in the brains of PD patients (Kia et al., 2021). These studies provide excellent examples of how multi-omics approaches combined with analyses of DNAm can be used to understand the impacts of genetic variants on the epigenome and transcriptome as well as to classify disease risk.

In addition to combining multiple types of omics data, several studies also explored single-omics network integration approaches in PD, using PD-associated candidate genes found in the literature or gene coexpression profiles to identify regulatory hubs (Liu et al., 2012; Chatterjee et al., 2017). Finally, global DNAm and DNAhm patterns in PD have been compared in the same individuals; however, it is still unclear how these two cytosine modifications interact at the site-specific level (Kaut et al., 2019). While the application of integrated multi-omics approaches toward understanding the pathogenesis of PD is still in its infancy, these recent studies provided promising evidence that leveraging multiple datasets can provide further insight regarding disease etiology than relying on one omics approach at a time.

## 7 Conclusion and future perspectives

Some of the greatest challenges in PD epigenetics research include complex interindividual etiology, disease heterogeneity, gradual progression, and the inaccessibility of human brain tissue to study the disease in real time. In addition, tissue culture and animal model studies cannot completely recapitulate human PD. However, the future of PD research is promising with the availability of a wide range of tools and resources to continue examining the etiological underpinnings and identify early-stage biomarkers of the disease. For example, analyses of patient cohorts in combination with model systems in the same study will facilitate the identification of loci associated with PD in humans, as well as providing a highly controlled setting in which to test the functionality of these variants and identify disease mechanisms. To address the issue of heterogeneity, experimental designs may also target associations with PD endophenotypes rather than taking a case–control approach. These two strategies have been successfully applied in a study of Alzheimer’s disease, where GWAS was used to identify variants associated with cerebrospinal fluid triggering receptor expressed on myeloid cells 2 (TREM2) levels, and tissue culture experiments were conducted to examine the functional implications of these variants (Deming et al., 2019). Secondary data analysis is also now possible due to the availability of large genomic databases, such as the UK Biobank, a prospective longitudinal study with detailed phenotyping that is estimated to include 14,000 individuals who will develop PD (Sudlow et al., 2015). The availability of large, well-phenotyped cohorts allows researchers to ask new questions regarding the etiology of PD as the field evolves. In addition, the breadth of integrated multi-omics methods and causal inference tools, such as Mendelian randomization, will aid in pinpointing gene regulatory networks related to disease. Overall, research in PD epigenomics will soon be limited only by the ability to ask pertinent research questions and apply creative analysis methods to uncover the “missing heritability” of this disorder.

## References

[B1] Aguilera O. Fernandez A. F. Muñoz A. Fraga M. F. (2010). Epigenetics and environment: A complex relationship. J. Appl. Physiology 109, 243–251. 10.1152/japplphysiol.00068.2010 20378707

[B2] Alfimova M. Kondratyev N. Golov A. Golimbet V. (2020). Profiling haplotype specific CpG and CpH methylation within a schizophrenia GWAS locus on chromosome 14 in schizophrenia and healthy subjects. Sci. Rep. 10, 4704. 10.1038/s41598-020-61671-2 32170143 PMC7069985

[B3] Angelopoulou E. Paudel Y. N. Papageorgiou S. G. Piperi C. (2022). Environmental impact on the epigenetic mechanisms underlying Parkinson's disease pathogenesis: A narrative review. Brain Sci. 12, 175. 10.3390/brainsci12020175 35203939 PMC8870303

[B4] Ascherio A. Schwarzschild M. A. (2016). The epidemiology of Parkinson's disease: Risk factors and prevention. Lancet Neurology 15, 1257–1272. 10.1016/S1474-4422(16)30230-7 27751556

[B5] Banovich N. E. Lan X. McVicker G. van de Geijn B. V. Degner J. F. Blischak J. D. (2014). Methylation QTLs are associated with coordinated changes in transcription factor binding, histone modifications, and gene expression levels. PLoS Genet. 10, e1004663. 10.1371/journal.pgen.1004663 25233095 PMC4169251

[B6] Barker D. J. (1990). The fetal and infant origins of adult disease. BMJ 301, 1111. 10.1136/bmj.301.6761.1111 2252919 PMC1664286

[B7] Barres R. Yan J. Egan B. Treebak J. T. Rasmussen M. Fritz T. (2012). Acute exercise remodels promoter methylation in human skeletal muscle. Cell Metab. 15, 405–411. 10.1016/j.cmet.2012.01.001 22405075

[B8] Betarbet R. Sherer T. B. MacKenzie G. Garcia-Osuna M. Panov A. V. Greenamyre J. T. (2000). Chronic systemic pesticide exposure reproduces features of Parkinson's disease. Nat. Neurosci. 3, 1301–1306. 10.1038/81834 11100151

[B9] Bibikova M. Le J. Barnes B. Saedinia-Melnyk S. Zhou L. Shen R. (2009). Genome-wide DNA methylation profiling using Infinium assay. Epigenomics 1, 177–200. 10.2217/epi.09.14 22122642

[B10] Bird A. (2007). Perceptions of epigenetics. Nature 447, 396–398. 10.1038/nature05913 17522671

[B11] Blanch M. Mosquera J. L. Ansoleaga B. Ferrer I. Barrachina M. (2016). Altered mitochondrial DNA methylation pattern in alzheimer disease-related pathology and in Parkinson disease. Am. J. Pathology 186, 385–397. 10.1016/j.ajpath.2015.10.004 26776077

[B12] Blauwendraat C. Nalls M. A. Singleton A. B. (2020). The genetic architecture of Parkinson's disease. Lancet Neurology 19, 170–178. 10.1016/s1474-4422(19)30287-x 31521533 PMC8972299

[B13] Booth M. J. Ost T. W. B. Beraldi D. Bell N. M. Branco M. R. Reik W. (2013). Oxidative bisulfite sequencing of 5-methylcytosine and 5-hydroxymethylcytosine. Nat. Protoc. 8, 1841–1851. 10.1038/nprot.2013.115 24008380 PMC3919000

[B14] Caldi Gomes L. Galhoz A. Jain G. Roser A.-E. Maass F. Carboni E. (2022). Multiomic landscaping of human midbrains identifies disease relevant molecular targets and pathways in advanced stage Parkinson's disease. Clin. Transl. Med 12, e692. 10.1002/ctm2.692 35090094 PMC8797064

[B15] Cavalli G. Heard E. (2019). Advances in epigenetics link genetics to the environment and disease. Nature 571, 489–499. 10.1038/s41586-019-1411-0 31341302

[B16] Cazaly E. Saad J. Wang W. Heckman C. Ollikainen M. Tang J. (2019). Making sense of the epigenome using data integration approaches. Front. Pharmacol. 10, 126. 10.3389/fphar.2019.00126 30837884 PMC6390500

[B17] Chang D. Nalls M. A. Nalls I. B. Hallgrímsdóttir J. Hunkapiller M. van der Brug F. (2017). A meta-analysis of genome-wide association studies identifies 17 new Parkinson's disease risk loci. Nat. Genet. 49, 1511–1516. 10.1038/ng.3955 28892059 PMC5812477

[B18] Chatterjee P. Roy D. Bhattacharyya M. Bandyopadhyay S. (2017). Biological networks in Parkinson's disease: An insight into the epigenetic mechanisms associated with this disease. BMC Genomics 18, 721. 10.1186/s12864-017-4098-3 28899360 PMC5596942

[B19] Chen H. Ritz B. (2018). The search for environmental causes of Parkinson's disease: Moving forward. Jpd 8, S9–S17. 10.3233/JPD-181493 30584168 PMC6311360

[B20] Chen H. Zhang S. M. Schwarzschild M. A. Hernan M. A. Ascherio A. (2005). Physical activity and the risk of Parkinson disease. Neurology 64, 664–669. 10.1212/01.WNL.0000151960.28687.93 15728289

[B21] Cheung W. A. Shao X. Morin A. Siroux V. Kwan T. Ge B. (2019). Correction to: Functional variation in allelic methylomes underscores a strong genetic contribution and reveals novel epigenetic alterations in the human epigenome. Genome Biol. 20, 89. 10.1186/s13059-019-1702-7 31064398 PMC6503438

[B22] Chiba-Falek O. Nussbaum R. L. (2001). Effect of allelic variation at the NACP-Rep1 repeat upstream of the alpha-synuclein gene (SNCA) on transcription in a cell culture luciferase reporter system. Hum. Mol. Genet. 10, 3101–3109. 10.1093/hmg/10.26.3101 11751692

[B23] Chuang Y.-H. Paul K. C. Bronstein J. M. Bordelon Y. Horvath S. Ritz B. (2017). Parkinson's disease is associated with DNA methylation levels in human blood and saliva. Genome Med. 9, 1–12. 10.1186/s13073-017-0466-5 28851441 PMC5576382

[B24] Coppede F. (2012). Genetics and epigenetics of Parkinson's disease. Sci. World J. 2012, 1–12. 10.1100/2012/489830 PMC3353471 22623900

[B25] Corti O. Lesage S. Brice A. (2011). What genetics tells us about the causes and mechanisms of Parkinson's disease. Physiol. Rev. 91, 1161–1218. 10.1152/physrev.00022.2010 22013209

[B26] Crotty G. F. Schwarzschild M. A. (2020). Chasing protection in Parkinson's disease: Does exercise reduce risk and progression? Front. Aging Neurosci. 12, 186. 10.3389/fnagi.2020.00186 32636740 PMC7318912

[B27] Curtis C. Shah S. P. Shah S.-F. Chin G. Turashvili O. M. Rueda M. J. (2012). The genomic and transcriptomic architecture of 2,000 breast tumours reveals novel subgroups. Nature 486, 346–352. 10.1038/nature10983 22522925 PMC3440846

[B28] Cutfield W. S. Hofman P. L. Mitchell M. Morison I. M. (2007). Could epigenetics play a role in the developmental origins of health and disease? Pediatr. Res. 61, 68R–75R. 10.1203/pdr.0b013e318045764c 17413843

[B29] Czamara D. Eraslan G. Eraslan C. M. Page J. Lahti M. Lahti-Pulkkinen E. (2019). Integrated analysis of environmental and genetic influences on cord blood DNA methylation in new-borns. Nat. Commun. 10, 2548. 10.1038/s41467-019-10461-0 31186427 PMC6559955

[B30] Darst R. P. Pardo C. E. Ai L. Brown K. D. Kladde M. P. (2010). Bisulfite sequencing of DNA. Curr. Protoc. Mol. Biol. 91, 7. 10.1002/0471142727.mb0709s91 PMC3214597 20583099

[B31] Davidi D. Schechter M. Elhadi S. A. Matatov A. Nathanson L. Sharon R. (2020). α-Synuclein translocates to the nucleus to activate retinoic-acid-dependent gene transcription. iScience 23, 100910. 10.1016/j.isci.2020.100910 32120069 PMC7052517

[B32] de Boni L. Riedel L. Schmitt I. Kraus T. F. J. Kaut O. Piston D. (2015). DNA methylation levels of α-synuclein intron 1 in the aging brain. Neurobiol. Aging 36, e7–3334. 10.1016/j.neurobiolaging.2015.08.028 26422361

[B33] Deaton A. M. Bird A. (2011). CpG islands and the regulation of transcription. Genes Dev. 25, 1010–1022. 10.1101/gad.2037511 21576262 PMC3093116

[B34] Delgado-Morales R. Esteller M. (2017). Opening up the DNA methylome of dementia. Mol. Psychiatry 22, 485–496. 10.1038/mp.2016.242 28044062 PMC5378809

[B35] Deming Y. Filipello F. Cignarella F. Cantoni C. Hsu S. Mikesell R. (2019). The MS4A gene cluster is a key modulator of soluble TREM2 and Alzheimer's disease risk. Sci. Transl. Med. 11, eaau2291. 10.1126/scitranslmed.aau2291 31413141 PMC6697053

[B36] Desplats P. Spencer B. Coffee E. Patel P. Michael S. Patrick C. (2011). α-Synuclein sequesters Dnmt1 from the nucleus. J. Biol. Chem. 286, 9031–9037. 10.1074/jbc.C110.212589 21296890 PMC3059002

[B37] Dumitriu A. Golji J. Labadorf A. T. Gao B. Beach T. G. Myers R. H. (2015). Integrative analyses of proteomics and RNA transcriptomics implicate mitochondrial processes, protein folding pathways and GWAS loci in Parkinson disease. BMC Med. Genomics 9, 5. 10.1186/s12920-016-0164-y PMC4722694 26793951

[B38] Dunn A. R. O’Connell K. M. S. Kaczorowski C. C. (2019). Gene-by-environment interactions in Alzheimer's disease and Parkinson's disease. Neurosci. Biobehav. Rev. 103, 73–80. 10.1016/j.neubiorev.2019.06.018 31207254 PMC6700747

[B39] Edgar R. D. Jones M. J. Meaney M. J. Turecki G. Kobor M. S. (2017). BECon: A tool for interpreting DNA methylation findings from blood in the context of brain. Transl. Psychiatry 7, e1187. 10.1038/tp.2017.171 28763057 PMC5611738

[B40] Elbaz A. Clavel J. Rathouz P. J. Moisan F. Galanaud J.-P. Delemotte B. (2009). Professional exposure to pesticides and Parkinson disease. Ann. Neurol. 66, 494–504. 10.1002/ana.21717 19847896

[B41] Elbaz A. Levecque C. Clavel J. Vidal J. S. Richard F. Amouyel P. (2004). CYP2D6 polymorphism, pesticide exposure, and Parkinson's disease. Ann. Neurol. 55, 430–434. 10.1002/ana.20051 14991823

[B42] Elsner V. R. Lovatel G. A. Moysés F. Bertoldi K. Spindler C. Cechinel L. R. (2013). Exercise induces age-dependent changes on epigenetic parameters in rat hippocampus: A preliminary study. Exp. Gerontol. 48, 136–139. 10.1016/j.exger.2012.11.011 23201423 PMC4405233

[B43] Emamzadeh F. N. (2016). Alpha-synuclein structure, functions, and interactions. J. Res. Med. Sci. 21, 29. 10.4103/1735-1995.181989 27904575 PMC5122110

[B44] Ernst J. Kellis M. (2012). ChromHMM: Automating chromatin-state discovery and characterization. Nat. Methods 9, 215–216. 10.1038/nmeth.1906 22373907 PMC3577932

[B45] Escott‐Price V. Nalls M. A. Morris H. R. Lubbe S. Brice A. Gasser T. (2015). Polygenic risk of P arkinson disease is correlated with disease age at onset. Ann. Neurol. 77, 582–591. 10.1002/ana.24335 25773351 PMC4737223

[B46] Espeso-Gil S. Holik A. Z. Bonnin S. Jhanwar S. Chandrasekaran S. Pique-Regi R. (2021). Environmental enrichment induces epigenomic and genome organization changes relevant for cognition. Front. Mol. Neurosci. 14, 664912. 10.3389/fnmol.2021.664912 34025350 PMC8131874

[B47] Farrer M. Wavrant-De Vrieze F. Crook R. Boles L. Perez-Tur J. Hardy J. (1998). Low frequency of ?-synuclein mutations in familial Parkinson's disease. Ann. Neurol. 43, 394–397. 10.1002/ana.410430320 9506559

[B48] Feinberg A. P. (2007). Phenotypic plasticity and the epigenetics of human disease. Nature 447, 433–440. 10.1038/nature05919 17522677

[B49] Fernandez-Santiago R. Carballo-Carbajal I. Castellano G. Torrent R. Richaud Y. Sánchez-Danes A. (2015). Aberrant epigenome in iPSC derived dopaminergic neurons from Parkinson's disease patients. EMBO Mol. Med. 7, 1529–1546. 10.15252/emmm.201505439 26516212 PMC4693505

[B50] Fernandez-Santiago R. Merkel A. Castellano G. Heath S. Raya A. Tolosa E. (2019). Whole-genome DNA hyper-methylation in iPSC-derived dopaminergic neurons from Parkinson's disease patients. Clin. Epigenet 11, 108. 10.1186/s13148-019-0701-6 PMC6651999 31337434

[B51] Fisher B. E. Wu A. D. Salem G. J. Song J. Lin C. H. J. Yip J. (2008). The effect of exercise training in improving motor performance and corticomotor excitability in people with early Parkinson's disease. Archives Phys. Med. Rehabilitation 89, 1221–1229. 10.1016/j.apmr.2008.01.013 PMC2989816 18534554

[B52] Freichel C. Neumann M. Ballard T. Müller V. Woolley M. Ozmen L. (2007). Age-dependent cognitive decline and amygdala pathology in α-synuclein transgenic mice. Neurobiol. Aging 28, 1421–1435. 10.1016/j.neurobiolaging.2006.06.013 16872721

[B53] Furlong M. A. Paul K. C. Yan Q. Chuang Y.-H. Cockburn M. G. Bronstein J. M. (2020). An epigenome-wide association study of ambient pyrethroid pesticide exposures in California's central valley. Int. J. Hyg. Environ. Health 229, 113569. 10.1016/j.ijheh.2020.113569 32679516 PMC7492449

[B54] Fuso A. Iyer A. M. van Scheppingen J. Maccarrone M. Scholl T. Hainfellner J. A. (2016). Promoter-specific hypomethylation correlates with IL-1β overexpression in tuberous sclerosis complex (TSC). J. Mol. Neurosci. 59, 464–470. 10.1007/s12031-016-0750-7 27122151 PMC4972849

[B55] Fuso A. Lucarelli M. (2019). CpG and non-CpG methylation in the diet-epigenetics-neurodegeneration connection. Curr. Nutr. Rep. 8, 74–82. 10.1007/s13668-019-0266-1 30887425

[B56] Gaunt T. R. Shihab H. A. Hemani G. Min J. L. Woodward G. Lyttleton O. (2016). Systematic identification of genetic influences on methylation across the human life course. Genome Biol. 17, 61. 10.1186/s13059-016-0926-z 27036880 PMC4818469

[B57] Gertz J. Varley K. E. Reddy T. E. Bowling K. M. Pauli F. Parker S. L. (2011). Analysis of DNA methylation in a three-generation family reveals widespread genetic influence on epigenetic regulation. PLoS Genet. 7, e1002228. 10.1371/journal.pgen.1002228 21852959 PMC3154961

[B58] Girirajan S. Rosenfeld J. A. Cooper G. M. Antonacci F. Siswara P. Itsara A. (2010). A recurrent 16p12.1 microdeletion supports a two-hit model for severe developmental delay. Nat. Genet. 42, 203–209. 10.1038/ng.534 20154674 PMC2847896

[B59] Go R. C. P. Corley M. J. Ross G. W. Petrovitch H. Masaki K. H. Maunakea A. K. (2020). Genome-wide epigenetic analyses in Japanese immigrant plantation workers with Parkinson's disease and exposure to organochlorines reveal possible involvement of glial genes and pathways involved in neurotoxicity. BMC Neurosci. 21, 1–18. 10.1186/s12868-020-00582-4 32650713 PMC7350633

[B60] Goldman S. M. Marek K. Ottman R. Meng C. Comyns K. Chan P. (2019). Concordance for Parkinson's disease in twins: A 20‐year update. Ann. Neurol. 85, 600–605. 10.1002/ana.25441 30786044

[B61] Gomez-Pinilla F. Zhuang Y. Feng J. Ying Z. Fan G. (2011). Exercise impacts brain-derived neurotrophic factor plasticity by engaging mechanisms of epigenetic regulation. Eur. J. Neurosci. 33, 383–390. 10.1111/j.1460-9568.2010.07508.x 21198979 PMC3256007

[B62] Gu H. Smith Z. D. Bock C. Boyle P. Gnirke A. Meissner A. (2011). Preparation of reduced representation bisulfite sequencing libraries for genome-scale DNA methylation profiling. Nat. Protoc. 6, 468–481. 10.1038/nprot.2010.190 21412275

[B63] Guintivano J. Aryee M. J. Kaminsky Z. A. (2013). A cell epigenotype specific model for the correction of brain cellular heterogeneity bias and its application to age, brain region and major depression. Epigenetics 8, 290–302. 10.4161/epi.23924 23426267 PMC3669121

[B64] Guo J. U. Su Y. Shin J. H. Shin J. Li H. Xie B. (2014). Distribution, recognition and regulation of non-CpG methylation in the adult mammalian brain. Nat. Neurosci. 17, 215–222. 10.1038/nn.3607 24362762 PMC3970219

[B65] Hack L. M. Dick A. L. W. Provencal N. (2016). Epigenetic mechanisms involved in the effects of stress exposure: Focus on 5-hydroxymethylcytosine: Table 1:. Environ. Epigenet. 2, dvw016. 10.1093/eep/dvw016 29492296 PMC5804530

[B66] He B. Zhang C. Zhang X. Fan Y. Zeng H. Liu J. (2021). Tissue-specific 5-hydroxymethylcytosine landscape of the human genome. Nat. Commun. 12, 4249. 10.1038/s41467-021-24425-w 34253716 PMC8275684

[B67] Henderson A. R. Wang Q. Meechoovet B. Siniard A. L. Naymik M. De Both M. (2021). DNA methylation and expression profiles of whole blood in Parkinson's disease. Front. Genet. 12, 640266. 10.3389/fgene.2021.640266 33981329 PMC8107387

[B68] Henderson-Smith A. Fisch K. M. Hua J. Liu G. Ricciardelli E. Jepsen K. (2019). DNA methylation changes associated with Parkinson's disease progression: Outcomes from the first longitudinal genome-wide methylation analysis in blood. Epigenetics 14, 365–382. 10.1080/15592294.2019.1588682 30871403 PMC6557551

[B69] Ho S.-M. Johnson A. Tarapore P. Janakiram V. Zhang X. Leung Y.-K. (2012). Environmental epigenetics and its implication on disease risk and health outcomes. ILAR J. 53, 289–305. 10.1093/ilar.53.3-4.289 23744968 PMC4021822

[B70] Horsthemke B. (2014). In brief: Genomic imprinting and imprinting diseases. J. Pathol. 232, 485–487. 10.1002/path.4326 24395592

[B71] Horvath S. Raj K. (2018). DNA methylation-based biomarkers and the epigenetic clock theory of ageing. Nat. Rev. Genet. 19, 371–384. 10.1038/s41576-018-0004-3 29643443

[B72] Husquin L. T. Rotival M. Fagny M. Quach H. Zidane N. McEwen L. M. (2018). Exploring the genetic basis of human population differences in DNA methylation and their causal impact on immune gene regulation. Genome Biol. 19, 222. 10.1186/s13059-018-1601-3 30563547 PMC6299574

[B73] Ihle J. Artaud F. Bekadar S. Mangone G. Sambin S. Mariani L. (2020). Parkinson's disease polygenic risk score is not associated with impulse control disorders: A longitudinal study. Park. Relat. Disord. 75, 30–33. 10.1016/j.parkreldis.2020.03.017 32450545

[B74] International Parkinson’s Disease Genomics Consortium and Wellcome Trust Case Control Consortium (2011). A two-stage meta-analysis identifies several new loci for Parkinson's disease. PLoS Genet. 7, e1002142. 10.1371/journal.pgen.1002142 21738488 PMC3128098

[B75] Inzelberg R. Schecthman E. Paleacu D. Zach L. Bonwitt R. Carasso R. l. (2004). Onset and progression of disease in familial and sporadic Parkinson's disease. Am. J. Med. Genet. 124A, 255–258. 10.1002/ajmg.a.20405 14708097

[B76] Irier H. Street R. C. Dave R. Lin L. Cai C. Davis T. H. (2014). Environmental enrichment modulates 5-hydroxymethylcytosine dynamics in hippocampus. Genomics 104, 376–382. 10.1016/j.ygeno.2014.08.019 25205305 PMC4252786

[B77] Irizarry R. A. Ladd-Acosta C. Wen B. Wu Z. Montano C. Onyango P. (2009). The human colon cancer methylome shows similar hypo- and hypermethylation at conserved tissue-specific CpG island shores. Nat. Genet. 41, 178–186. 10.1038/ng.298 19151715 PMC2729128

[B78] Islam S. A. Goodman S. J. MacIsaac J. L. Obradovic J. Barr R. G. Boyce W. T. (2019). Integration of DNA methylation patterns and genetic variation in human pediatric tissues help inform EWAS design and interpretation. Epigenetics Chromatin 12, 1. 10.1186/s13072-018-0245-6 30602389 PMC6314079

[B79] Jessop P. Toledo-Rodriguez M. (2018). Hippocampal TET1 and TET2 expression and DNA hydroxymethylation are affected by physical exercise in aged mice. Front. Cell Dev. Biol. 6, 45. 10.3389/fcell.2018.00045 29732371 PMC5922180

[B80] Jin H. Kanthasamy A. Ghosh A. Yang Y. Anantharam V. Kanthasamy A. G. (2011). -synuclein negatively regulates protein kinase C expression to suppress apoptosis in dopaminergic neurons by reducing p300 histone acetyltransferase activity. J. Neurosci. 31, 2035–2051. 10.1523/JNEUROSCI.5634-10.2011 21307242 PMC3041642

[B81] Jo E. Fuller N. Rand R. P. St George-Hyslop P. Fraser P. E. (2002). Defective membrane interactions of familial Parkinson's disease mutant A30P α-synuclein 1 1Edited by I. B. Holland. J. Mol. Biol. 315, 799–807. 10.1006/jmbi.2001.5269 11812148

[B82] Jones M. J. Goodman S. J. Kobor M. S. (2015). DNAmethylation and healthy human aging. Aging Cell 14, 924–932. 10.1111/acel.12349 25913071 PMC4693469

[B83] Jones P. A. (2012). Functions of DNA methylation: Islands, start sites, gene bodies and beyond. Nat. Rev. Genet. 13, 484–492. 10.1038/nrg3230 22641018

[B84] Jowaed A. Schmitt I. Kaut O. Wullner U. (2010). Methylation regulates alpha-synuclein expression and is decreased in Parkinson's disease patients' brains. J. Neurosci. 30, 6355–6359. 10.1523/JNEUROSCI.6119-09.2010 20445061 PMC6632710

[B85] Kaas G. A. Zhong C. Eason D. E. Ross D. L. Vachhani R. V. Ming G.-L. (2013). TET1 controls CNS 5-methylcytosine hydroxylation, active DNA demethylation, gene transcription, and memory formation. Neuron 79, 1086–1093. 10.1016/j.neuron.2013.08.032 24050399 PMC3816951

[B86] Kantor B. Tagliafierro L. Gu J. Zamora M. E. Ilich E. Grenier C. (2018). Downregulation of SNCA expression by targeted editing of DNA methylation: A potential strategy for precision therapy in PD. Mol. Ther. 26, 2638–2649. 10.1016/j.ymthe.2018.08.019 30266652 PMC6224806

[B87] Kaushal A. Zhang H. Karmaus W. J. J. Ray M. Torres M. A. Smith A. K. (2017). Comparison of different cell type correction methods for genome-scale epigenetics studies. BMC Bioinforma. 18, 216. 10.1186/s12859-017-1611-2 PMC5391562 28410574

[B88] Kaut O. Kuchelmeister K. Moehl C. Wullner U. (2019). 5-methylcytosine and 5-hydroxymethylcytosine in brains of patients with multiple system atrophy and patients with Parkinson's disease. J. Chem. Neuroanat. 96, 41–48. 10.1016/j.jchemneu.2018.12.005 30557654

[B89] Kaut O. Schmitt I. Stahl F. Fröhlich H. Hoffmann P. Gonzalez F. J. (2022). Epigenome-wide analysis of DNA methylation in Parkinson's disease cortex. Life 12, 502. 10.3390/life12040502 35454993 PMC9025601

[B90] Kaut O. Schmitt I. Wüllner U. (2012). Genome-scale methylation analysis of Parkinson's disease patients' brains reveals DNA hypomethylation and increased mRNA expression of cytochrome P450 2E1. Neurogenetics 13, 87–91. 10.1007/s10048-011-0308-3 22238121

[B91] Kernaleguen M. Daviaud C. Shen Y. Bonnet E. Renault V. Deleuze J.-F. (2018). “Whole-genome bisulfite sequencing for the analysis of genome-wide DNA methylation and hydroxymethylation patterns at single-nucleotide resolution,” in Epigenome editing: Methods and protocols methods in molecular biology. Editors Jeltsch A. Rots M. G. (New York, NY: Springer), 311–349. 10.1007/978-1-4939-7774-1_18 29524144

[B92] Kia D. A. Zhang D. Guelfi S. Manzoni C. Hubbard L. Reynolds R. H. (2021). Identification of candidate Parkinson disease genes by integrating genome-wide association study, expression, and epigenetic data sets. JAMA Neurol. 78, 464–472. 10.1001/jamaneurol.2020.5257 33523105 PMC7851759

[B93] Kim S. Xing E. P. (2012). Tree-guided group lasso for multi-response regression with structured sparsity, with an application to eQTL mapping. Ann. Appl. Stat. 6, 1095–1117. 10.1214/12-aoas549

[B94] Kinde B. Gabel H. W. Gilbert C. S. Griffith E. C. Greenberg M. E. (2015). Reading the unique DNA methylation landscape of the brain: Non-CpG methylation, hydroxymethylation, and MeCP2. Proc. Natl. Acad. Sci. U.S.A. 112, 6800–6806. 10.1073/pnas.1411269112 25739960 PMC4460470

[B95] Klein C. Westenberger A. (2012). Genetics of Parkinson's disease. Cold Spring Harb. Perspect. Med. 2, a008888. 10.1101/cshperspect.a008888 22315721 PMC3253033

[B96] Kochmanski J. Bernstein A. I. (2020). The impact of environmental factors on 5-hydroxymethylcytosine in the brain. Curr. Envir Health Rpt 7, 109–120. 10.1007/s40572-020-00268-3 PMC7809708 32020534

[B97] Kontopoulos E. Parvin J. D. Feany M. B. (2006). α-synuclein acts in the nucleus to inhibit histone acetylation and promote neurotoxicity. Hum. Mol. Genet. 15, 3012–3023. 10.1093/hmg/ddl243 16959795

[B98] Krueger F. Andrews S. R. (2012). Quality control, trimming and alignment of Bisulfite-Seq data. AvaliableAt: https://www.epigenesys.eu/images/stories/protocols/pdf/20120720103700_p57.pdf (Accessed June 14, 2022).

[B99] Kumar S. Chinnusamy V. Mohapatra T. (2018). Epigenetics of modified DNA bases: 5-methylcytosine and beyond. Front. Genet. 9, 640. 10.3389/fgene.2018.00640 30619465 PMC6305559

[B100] Laird P. W. (2010). Principles and challenges of genome-wide DNA methylation analysis. Nat. Rev. Genet. 11, 191–203. 10.1038/nrg2732 20125086

[B102] Lang A. E. Lozano A. M. (1998). Parkinson's disease. N. Engl. J. Med. 339, 1044–1053. 10.1056/NEJM199810083391506 9761807

[B103] Langston J. W. Ballard P. Tetrud J. W. Irwin I. (1983). Chronic parkinsonism in humans due to a product of meperidine-analog synthesis. Science 219, 979–980. 10.1126/science.6823561 6823561

[B104] Lappalainen T. Greally J. M. (2017). Associating cellular epigenetic models with human phenotypes. Nat. Rev. Genet. 18, 441–451. 10.1038/nrg.2017.32 28555657

[B105] Lee J.-H. Saito Y. Park S.-J. Nakai K. (2020). Existence and possible roles of independent non-CpG methylation in the mammalian brain. DNA Res. 27, dsaa020. 10.1093/dnares/dsaa020 32970817 PMC7750974

[B106] Lee K. W. K. Pausova Z. (2013). Cigarette smoking and DNA methylation. Front. Genet. 4, 132. 10.3389/fgene.2013.00132 23882278 PMC3713237

[B107] Lesage S. Brice A. (2009). Parkinson's disease: From monogenic forms to genetic susceptibility factors. Hum. Mol. Genet. 18, R48–R59. 10.1093/hmg/ddp012 19297401

[B108] Levine M. E. Lu A. T. Quach A. Chen B. H. Assimes T. L. Bandinelli S. (2018). An epigenetic biomarker of aging for lifespan and healthspan. Aging 10, 573–591. 10.18632/aging.101414 29676998 PMC5940111

[B109] Li P. Ensink E. Lang S. Marshall L. Schilthuis M. Lamp J. (2020). Hemispheric asymmetry in the human brain and in Parkinson's disease is linked to divergent epigenetic patterns in neurons. Genome Biol. 21, 1–23. 10.1186/s13059-020-01960-1 PMC7063821 32151270

[B110] Li X. Wei W. Zhao Q.-Y. Widagdo J. Baker-Andresen D. Flavell C. R. (2014). Neocortical Tet3-mediated accumulation of 5-hydroxymethylcytosine promotes rapid behavioral adaptation. Proc. Natl. Acad. Sci. U.S.A. 111, 7120–7125. 10.1073/pnas.1318906111 24757058 PMC4024925

[B111] Li Y. Ma L. Wu D. Chen G. (2021). Advances in bulk and single-cell multi-omics approaches for systems biology and precision medicine. Brief. Bioinform. 22, bbab024. 10.1093/bib/bbab024 33778867

[B112] Lim U. Song M.-A. (2012). “Dietary and lifestyle factors of DNA methylation,” in Cancer epigenetics: Methods and protocols methods in molecular biology. Editors Dumitrescu R. G. Verma M. (Totowa, NJ: Humana Press), 359–376. 10.1007/978-1-61779-612-8_23 22359306

[B113] Lin M. K. Farrer M. J. (2014). Genetics and genomics of Parkinson's disease. Genome Med. 6, 48. 10.1186/gm566 25061481 PMC4085542

[B114] Lindholm M. E. Marabita F. Gomez-Cabrero D. Rundqvist H. Ekström T. J. Tegnér J. (2014). An integrative analysis reveals coordinated reprogramming of the epigenome and the transcriptome in human skeletal muscle after training. Epigenetics 9, 1557–1569. 10.4161/15592294.2014.982445 25484259 PMC4622000

[B115] Lister R. Mukamel E. A. Nery J. R. Urich M. Puddifoot C. A. Johnson N. D. (2013). Global epigenomic reconfiguration during mammalian brain development. Science 341, 1237905. 10.1126/science.1237905 23828890 PMC3785061

[B116] Liu J. Lin D. Chen J. Perrone-Bizzozero N. Calhoun V. Bustillo J. (2019). Characterization of cross-tissue mQTL effects and their relevance in psychiatric disorders. Eur. Neuropsychopharmacol. 29, S796–S797. 10.1016/j.euroneuro.2017.08.030

[B117] Liu J. Siegmund K. D. (2016). An evaluation of processing methods for HumanMethylation450 BeadChip data. BMC Genomics 17, 469. 10.1186/s12864-016-2819-7 27334613 PMC4918139

[B118] Liu Y. Koyutürk M. Maxwell S. Zhao Z. Chance M. R. (2012). Integrative analysis of common neurodegenerative diseases using gene association, interaction networks and mRNA expression data. AMIA Jt. Summits Transl. Sci. Proc. 2012, 62–71. 22779053 PMC3392058

[B119] Lock E. F. Dunson D. B. (2013). Bayesian consensus clustering. Bioinformatics 29, 2610–2616. 10.1093/bioinformatics/btt425 23990412 PMC3789539

[B120] Logroscino G. (2005). The role of early life environmental risk factors in Parkinson disease: What is the evidence? Environ. Health Perspect. 113, 1234–1238. 10.1289/ehp.7573 16140634 PMC1280408

[B121] Lokk K. Modhukur V. Rajashekar B. Märtens K. Mägi R. Kolde R. (2016). Erratum to: DNA methylome profiling of human tissues identifies global and tissue-specific methylation patterns. Genome Biol. 17, 224. 10.1186/s13059-016-1091-0 27802833 PMC5090885

[B122] Lunnon K. Hannon E. G.Smith R. G. Dempster E. Wong C. Burrage J. (2016). Erratum to: Variation in 5-hydroxymethylcytosine across human cortex and cerebellum. Genome BiolGenome Biol. 1717, 13127. 10.1186/s13059-016-0958-410.1186/s13059-016-0871-x PMC4756397 26883014

[B123] Maraganore D. M. de Andrade M. Elbaz A. Farrer M. J. Ioannidis J. P. Krüger R. (2006). Collaborative analysis of α-synuclein gene promoter variability and Parkinson disease. JAMA 296, 661–670. 10.1001/jama.296.6.661 16896109

[B124] Marey-Semper I. Gelman M. Levi-Strauss M. (1995). A selective toxicity toward cultured mesencephalic dopaminergic neurons is induced by the synergistic effects of energetic metabolism impairment and NMDA receptor activation. J. Neurosci. 15, 5912–5918. 10.1523/jneurosci.15-09-05912.1995 7666176 PMC6577653

[B125] Marshall L. L. Killinger B. A. Ensink E. Li P. Li K. X. Cui W. (2020). Epigenomic analysis of Parkinson's disease neurons identifies Tet2 loss as neuroprotective. Nat. Neurosci. 23, 1203–1214. 10.1038/s41593-020-0690-y 32807949

[B126] Martins M. Rosa A. Guedes L. C. Fonseca B. V. Gotovac K. Violante S. (2011). Convergence of miRNA expression profiling, α-synuclein interacton and GWAS in Parkinson's disease. PLoS ONE 6, e25443. 10.1371/journal.pone.0025443 22003392 PMC3189215

[B127] Marttinen P. Pirinen M. Sarin A.-P. Gillberg J. Kettunen J. Surakka I. (2014). Assessing multivariate gene-metabolome associations with rare variants using Bayesian reduced rank regression. Bioinformatics 30, 2026–2034. 10.1093/bioinformatics/btu140 24665129 PMC4080737

[B128] Maserejian N. Vinikoor-Imler L. Dilley A. (2020). Estimation of the 2020 global population of Parkinson’s disease (PD). Available at: https://www.mdsabstracts.org/abstract/estimation-of-the-2020-global-population-of-parkinsons-disease-pd/ (Accessed June 14, 2022).

[B129] Masliah E. Dumaop W. Galasko D. Desplats P. (2013). Distinctive patterns of DNA methylation associated with Parkinson disease. Epigenetics 8, 1030–1038. 10.4161/epi.25865 23907097 PMC3891683

[B130] Matosin N. Cruceanu C. Binder E. B. (2017). Preclinical and clinical evidence of DNA methylation changes in response to trauma and chronic stress. Chronic Stress 1, 247054701771076. 10.1177/2470547017710764 PMC5831952 29503977

[B131] Maver A. Peterlin B. (2011). Positional integratomic approach in identification of genomic candidate regions for Parkinson's disease. Bioinformatics 27, 1971–1978. 10.1093/bioinformatics/btr313 21596793

[B132] McCormack A. L. Thiruchelvam M. Manning-Bog A. B. Thiffault C. Langston J. W. Cory-Slechta D. A. (2002). Environmental risk factors and Parkinson's disease: Selective degeneration of nigral dopaminergic neurons caused by the herbicide paraquat. Neurobiol. Dis. 10, 119–127. 10.1006/nbdi.2002.0507 12127150

[B133] Moore K. McKnight A. J. Craig D. O’Neill F. (2014). Epigenome-wide association study for Parkinson's disease. Neuromol. Med. 16, 845–855. 10.1007/s12017-014-8332-8 25304910

[B134] Multhaup M. L. Seldin M. Jaffe A. E. Lei X. Kirchner H. Mondal P. (2015). Mouse-human experimental epigenetic analysis unmasks dietary targets and genetic liability for diabetic phenotypes. Cell Metab. 21, 138–149. 10.1016/j.cmet.2014.12.014 25565211 PMC4340475

[B135] Muñoz E. Oliva R. Obach V. Marti M. J. Pastor P. Ballesta F. (1997). Identification of Spanish familial Parkinson’s disease and screening for the Ala53Thr mutation of the α-synuclein gene in early onset patients. Neurosci. Lett. 235, 57–60. 10.1016/S0304-3940(97)00710-6 9389595

[B136] Nabais M. F. Laws S. M. Laws T. Lin C. L. Vallerga N. J. Armstrong I. P. (2021). Meta-analysis of genome-wide DNA methylation identifies shared associations across neurodegenerative disorders. Genome Biol. 22, 1–30. 10.1186/s13059-021-02275-5 33771206 PMC8004462

[B137] Nalls M. A. Blauwendraat C. Vallerga C. L. Heilbron K. Bandres-Ciga S. Chang D. (2019). Identification of novel risk loci, causal insights, and heritable risk for Parkinson's disease: A meta-analysis of genome-wide association studies. Lancet Neurol. 18, 1091–1102. 10.1016/S1474-4422(19)30320-5 31701892 PMC8422160

[B138] Nalls M. A. Pankratz N. Pankratz C. M. Lill C. B. Do D. G. Hernandez M. (2014). Large-scale meta-analysis of genome-wide association data identifies six new risk loci for Parkinson's disease. Nat. Genet. 46, 989–993. 10.1038/ng.3043 25064009 PMC4146673

[B139] Ngwa J. S. Nwulia E. Ntekim O. Bedada F. B. Kwabi-Addo B. Nadarajah S. (2022). Aerobic exercise training-induced changes on DNA methylation in mild cognitively impaired elderly african Americans: Gene, exercise, and memory study - GEMS-I. Front. Mol. Neurosci. 14, 752403. 10.3389/fnmol.2021.752403 35110995 PMC8802631

[B140] Nicolia V. Cavallaro R. A. Lopez-Gonzalez I. Maccarrone M. Scarpa S. Ferrer I. (2017). DNA methylation profiles of selected pro-inflammatory cytokines in Alzheimer disease. J. Neuropathol. Exp. Neurol. 76, nlw099–31. 10.1093/jnen/nlw099 28053004

[B141] Oliveira L. M. A. Falomir-Lockhart L. J. Botelho M. G. Lin K.-H. Wales P. Koch J. C. (2015). Elevated α-synuclein caused by SNCA gene triplication impairs neuronal differentiation and maturation in Parkinson's patient-derived induced pluripotent stem cells. Cell Death Dis. 6, e1994. 10.1038/cddis.2015.318 26610207 PMC4670926

[B142] Paiva I. Jain G. Lazaro D. F. Jercic K. G. Hentrich T. Kerimoglu C. (2018). Alpha-synuclein deregulates the expression of COL4A2 and impairs ER-Golgi function. Neurobiol. Dis. 119, 121–135. 10.1016/j.nbd.2018.08.001 30092270

[B143] Paiva I. Pinho R. Pavlou M. A. Hennion M. Wales P. Schütz A.-L. (2017). Sodium butyrate rescues dopaminergic cells from alpha-synuclein-induced transcriptional deregulation and DNA damage. Hum. Mol. Genet. 26, 2231–2246. 10.1093/hmg/ddx114 28369321

[B144] Pang S. Y.-Y. Ho P. W.-L. Liu H.-F. Leung C.-T. Li L. Chang E. E. S. (2019). The interplay of aging, genetics and environmental factors in the pathogenesis of Parkinson's disease. Transl. Neurodegener. 8, 1–11. 10.1186/s40035-019-0165-9 31428316 PMC6696688

[B145] Park G. Tan J. Garcia G. Kang Y. Salvesen G. Zhang Z. (2016). Regulation of histone acetylation by autophagy in Parkinson disease. J. Biol. Chem. 291, 3531–3540. 10.1074/jbc.M115.675488 26699403 PMC4751393

[B146] Peng J. Mao X. O. Stevenson F. F. Hsu M. Andersen J. K. (2004). The herbicide paraquat induces dopaminergic nigral apoptosis through sustained activation of the JNK pathway. J. Biol. Chem. 279, 32626–32632. 10.1074/jbc.M404596200 15155744

[B147] Peterlin B. Maver A. (2012). Integrative 'omic' approach towards understanding the nature of human diseases. Balk. J. Med. Genet. 15, 45–50. 10.2478/v10034-012-0018-7 PMC3776674 24052743

[B148] Pidsley R. Y Wong C. C. Volta M. Lunnon K. Mill J. Schalkwyk L. C. (2013). A data-driven approach to preprocessing Illumina 450K methylation array data. BMC Genomics 14, 293. 10.1186/1471-2164-14-293 23631413 PMC3769145

[B149] Pidsley R. Zotenko E. Peters T. J. Lawrence M. G. Risbridger G. P. Molloy P. (2016). Critical evaluation of the Illumina MethylationEPIC BeadChip microarray for whole-genome DNA methylation profiling. Genome Biol. 17, 208. 10.1186/s13059-016-1066-1 27717381 PMC5055731

[B150] Pinho R. Paiva I. Jercic K. G. Fonseca-Ornelas L. Gerhardt E. Fahlbusch C. (2019). Nuclear localization and phosphorylation modulate pathological effects of alpha-synuclein. Hum. Mol. Genet. 28, 31–50. 10.1093/hmg/ddy326 30219847

[B151] Polymeropoulos M. H. Higgins J. J. Golbe L. I. Johnson W. G. Ide S. E. Di Iorio G. (1996). Mapping of a gene for Parkinson's disease to chromosome 4q21-q23. Science 274, 1197–1199. 10.1126/science.274.5290.1197 8895469

[B152] Polymeropoulos M. H. Lavedan C. Leroy E. Ide S. E. Dehejia A. Dutra A. (1997). Mutation in the α-synuclein gene identified in families with Parkinson's disease. Science 276, 2045–2047. 10.1126/science.276.5321.2045 9197268

[B153] Pouchieu C. Piel C. Carles C. Gruber A. Helmer C. Tual S. (2018). Pesticide use in agriculture and Parkinson's disease in the AGRICAN cohort study. Int. J. Epidemiol. 47, 299–310. 10.1093/ije/dyx225 29136149

[B154] Przedborski S. Jackson-Lewis V. Naini A. B. Jakowec M. Petzinger G. Miller R. (2001). The parkinsonian toxin 1-methyl-4-phenyl-1,2,3,6-tetrahydropyridine (MPTP): A technical review of its utility and safety. J. Neurochem. 76, 1265–1274. 10.1046/j.1471-4159.2001.00183.x 11238711

[B155] Ronn T. Volkov P. Davegardh C. Dayeh T. Hall E. Olsson A. H. (2013). A six months exercise intervention influences the genome-wide DNA methylation pattern in human adipose tissue. PLoS Genet. 9, e1003572. 10.1371/journal.pgen.1003572 23825961 PMC3694844

[B156] Rajput A. H. Uitti R. J. Stern W. Laverty W. O'Donnell K. O'Donnell D. (1987). Geography, drinking water chemistry, pesticides and herbicides and the etiology of Parkinson's disease. Can. J. Neurol. Sci. 14, 414–418. 10.1017/S0317167100037823 3676917

[B157] Rawlik K. Rowlatt A. Tenesa A. (2016). Imputation of DNA methylation levels in the brain implicates a risk factor for Parkinson's disease. Genetics 204, 771–781. 10.1534/genetics.115.185967 27466229 PMC5068861

[B158] Rawson K. S. McNeely M. E. Duncan R. P. Pickett K. A. Perlmutter J. S. Earhart G. M. (2019). Exercise and Parkinson disease: Comparing tango, treadmill, and stretching. J. Neurol. Phys. Ther. 43, 26–32. 10.1097/NPT.0000000000000245 30531383 PMC6294320

[B159] Redensek S. Dolzan V. Kunej T. (2018). From genomics to omics landscapes of Parkinson's disease: Revealing the molecular mechanisms. OMICS A J. Integr. Biol. 22, 1–16. 10.1089/omi.2017.0181 PMC5784788 29356624

[B160] Reimand J. Isserlin R. Voisin V. Kucera M. Tannus-Lopes C. Rostamianfar A. (2019). Pathway enrichment analysis and visualization of omics data using g:Profiler, GSEA, Cytoscape and EnrichmentMap. Nat. Protoc. 14, 482–517. 10.1038/s41596-018-0103-9 30664679 PMC6607905

[B161] Richardson S. Tseng G. C. Sun W. (2016). Statistical methods in integrative genomics. Annu. Rev. Stat. Appl. 3, 181–209. 10.1146/annurev-statistics-041715-033506 27482531 PMC4963036

[B162] Ritz B. R. Manthripragada A. D. Costello S. Lincoln S. J. Farrer M. J. Cockburn M. (2009). Dopamine transporter genetic variants and pesticides in Parkinson's disease. Environ. Health Perspect. 117, 964–969. 10.1289/ehp.0800277 19590691 PMC2702414

[B163] Rudyk C. Dwyer Z. Dwyer S. Hayley C. (2019). Leucine-rich repeat kinase-2 (LRRK2) modulates paraquat-induced inflammatory sickness and stress phenotype. J. Neuroinflammation 16, 120. 10.1186/s12974-019-1483-7 31174552 PMC6554960

[B164] Russo V. E. A. Martienssen R. A. Riggs A. D. (1996). Epigenetic mechanisms of gene regulation. Plainview, N.Y: Cold Spring Harbor Laboratory Press.

[B165] Swiatowy W. J. Drzewiecka H. Kliber M. Sąsiadek M. Karpinski P. Pławski A. (2021). Physical activity and DNA methylation in humans. Ijms 22, 12989. 10.3390/ijms222312989 34884790 PMC8657566

[B166] Sasco A. J. Paffenbarger R. S. Gendre I. Wing A. L. (1992). The role of physical exercise in the occurrence of Parkinson's disease. Archives Neurology 49, 360–365. 10.1001/archneur.1992.00530280040020 1558515

[B167] Schaffner S. L. Wassouf Z. Lazaro D. F. Xylaki M. Gladish N. Lin D. T. S. (2022). Alpha-synuclein overexpression induces epigenomic dysregulation of glutamate signaling and locomotor pathways. Hum. Mol. Genet., ddac104. 10.1093/hmg/ddac104 35567546 PMC9616577

[B168] Schmitt I. Kaut O. Khazneh H. deBoni L. Ahmad A. Berg D. (2015). L-dopa increases α -synuclein DNA methylation in Parkinson's disease patients *in vivo* and *in vitro* . Mov. Disord. 30, 1794–1801. 10.1002/mds.26319 26173746

[B169] Scott W. K. Stajich J. M. Yamaoka L. H. Speer M. C. Vance J. M. Roses A. D. (1997). Genetic complexity and Parkinson's disease. Science 277, 387–390. 10.1126/science.277.5324.387 9518366

[B170] Scott W. K. Yamaoka L. H. Stajich J. M. Scott B. L. Vance J. M. Roses A. D. (1999). The α-synuclein gene is not a major risk factor in familial Parkinson disease. Neurogenetics 2, 191–192. 10.1007/s100480050083 10541595

[B171] Shi X. Radhakrishnan S. Wen J. Chen J. Y. Chen J. Lam B. A. (2020). Association of CNVs with methylation variation. NPJ Genom. Med. 5, 41. 10.1038/s41525-020-00145-w 33062306 PMC7519119

[B172] Singh A. Shannon C. P. Gautier B. Rohart F. Vacher M. Tebbutt S. J. (2019). Diablo: An integrative approach for identifying key molecular drivers from multi-omics assays. Bioinformatics 35, 3055–3062. 10.1093/bioinformatics/bty1054 30657866 PMC6735831

[B173] Skvortsova K. Zotenko E. Luu P.-L. Gould C. M. Nair S. S. Clark S. J. (2017). Comprehensive evaluation of genome-wide 5-hydroxymethylcytosine profiling approaches in human DNA. Epigenetics Chromatin 10, 16. 10.1186/s13072-017-0123-7 28428825 PMC5397694

[B174] Slieker R. C. van Iterson M. van Iterson R. Luijk M. Beekman D. V. Zhernakova M. H. (2016). Age-related accrual of methylomic variability is linked to fundamental ageing mechanisms. Genome Biol. 17, 191. 10.1186/s13059-016-1053-6 27654999 PMC5032245

[B175] Smith A. K. Kilaru V. Kocak M. Almli L. M. Mercer K. B. Ressler K. J. (2014). Methylation quantitative trait loci (meQTLs) are consistently detected across ancestry, developmental stage, and tissue type. BMC Genomics 15, 145. 10.1186/1471-2164-15-145 24555763 PMC4028873

[B176] Spiers H. Hannon E. Schalkwyk L. C. Bray N. J. Mill J. (2017). 5-hydroxymethylcytosine is highly dynamic across human fetal brain development. BMC Genomics 18, 738. 10.1186/s12864-017-4091-x 28923016 PMC5604137

[B177] Stingo F. C. Chen Y. A. Tadesse M. G. Vannucci M. (2011). Incorporating biological information into linear models: A bayesian approach to the selection of pathways and genes. Ann. Appl. Stat. 5, 463. 10.1214/11-AOAS46310.1214/11-AOAS463 PMC3650864 23667412

[B178] Stricker S. H. Gotz M. (2018). DNA-Methylation: Master or slave of neural fate decisions? Front. Neurosci. 12, 5. 10.3389/fnins.2018.00005 29449798 PMC5799221

[B179] Sudlow C. Gallacher J. Allen N. Beral V. Burton P. Danesh J. (2015). UK Biobank: An open access resource for identifying the causes of a wide range of complex diseases of middle and old age. PLoS Med. 12, e1001779. 10.1371/journal.pmed.1001779 25826379 PMC4380465

[B180] Sujkowski A. Hong L. Wessells R. J. Todi S. V. (2022). The protective role of exercise against age-related neurodegeneration. Ageing Res. Rev. 74, 101543. 10.1016/j.arr.2021.101543 34923167 PMC8761166

[B181] Szulwach K. E. Li X. Li Y. Song C.-X. Wu H. Dai Q. (2011). 5-hmC-mediated epigenetic dynamics during postnatal neurodevelopment and aging. Nat. Neurosci. 14, 1607–1616. 10.1038/nn.2959 22037496 PMC3292193

[B182] Tanner C. M. Kamel F. Ross G. W. Hoppin J. A. Goldman S. M. Korell M. (2011). Rotenone, paraquat, and Parkinson's disease. Environ. Health Perspect. 119, 866–872. 10.1289/ehp.1002839 21269927 PMC3114824

[B183] Teh A. L. Pan H. Chen L. Ong M.-L. Dogra S. Wong J. (2014). The effect of genotype and *in utero* environment on interindividual variation in neonate DNA methylomes. Genome Res. 24, 1064–1074. 10.1101/gr.171439.113 24709820 PMC4079963

[B184] Tiili E. M. Antikainen M. S. H. Mitiushkina N. V. Sukhovskaya O. A. Imyanitov E. N. Hirvonen A. P. (2015). Effect of genotype and methylation of CYP2D6 on smoking behaviour. Pharmacogenet. Genomics 25, 531–540. 10.1097/FPC.0000000000000166 26287939

[B185] Vallerga C. L. Zhang F. Fowdar J. McRae A. F. Qi T. Nabais M. F. (2020). Analysis of DNA methylation associates the cystine-glutamate antiporter SLC7A11 with risk of Parkinson's disease. Nat. Commun. 11, 1238. 10.1038/s41467-020-15065-7 32144264 PMC7060318

[B186] van der Plaat D. A. de Jong K. de Vries M. van Diemen C. C. Nedeljkovic I. Amin N. (2018). Occupational exposure to pesticides is associated with differential DNA methylation. Occup. Environ. Med. 75, 427–435. 10.1136/oemed-2017-104787 29459480 PMC5969365

[B187] Villar-Piqué A. Lopes da Fonseca T. Outeiro T. F. (2016). Structure, function and toxicity of alpha-synuclein: The Bermuda triangle in synucleinopathies. J. Neurochem. 139, 240–255. 10.1111/jnc.13249 26190401

[B188] Villicana S. Bell J. T. (2021). Genetic impacts on DNA methylation: Research findings and future perspectives. Genome Biol. 22, 127. 10.1186/s13059-021-02347-6 33931130 PMC8086086

[B189] Voutsinas G. E. Stavrou E. F. Karousos G. Dasoula A. Papachatzopoulou A. Syrrou M. (2010). Allelic imbalance of expression and epigenetic regulation within the alpha-synuclein wild-type and p.Ala53Thr alleles in Parkinson disease. Hum. Mutat. 31, 685–691. 10.1002/humu.21248 20340137

[B190] Waddington C. H. (1957). The strategy of the genes. London: Routledge. 10.4324/9781315765471

[B191] Wang C. Chen L. Yang Y. Zhang M. Wong G. (2019b). Identification of potential blood biomarkers for Parkinson's disease by gene expression and DNA methylation data integration analysis. Clin. Epigenet. 11, 1–15. 10.1186/s13148-019-0621-5 PMC6371578 30744671

[B192] Wang H. Lou D. Wang Z. (2019a). Crosstalk of genetic variants, allele-specific DNA methylation, and environmental factors for complex disease risk. Front. Genet. 9, 1–15. 10.3389/fgene.2018.00695 PMC6334214 30687383

[B193] Wassouf Z. Hentrich T. Samer S. Rotermund C. Kahle P. J. Ehrlich I. (2018). Environmental enrichment prevents transcriptional disturbances induced by alpha-synuclein overexpression. Front. Cell. Neurosci. 12, 112. 10.3389/fncel.2018.00112 29755323 PMC5932345

[B194] Wassouf Z. Schulze-Hentrich J. M. (2019). Alpha-synuclein at the nexus of genes and environment: The impact of environmental enrichment and stress on brain health and disease. J. Neurochem. 150, 591–604. 10.1111/jnc.14787 31165472 PMC6771760

[B195] Wen L. Li X. Yan L. Tan Y. Li R. Zhao Y. (2014). Whole-genome analysis of 5-hydroxymethylcytosine and 5-methylcytosine at base resolution in the human brain. Genome Biol. 15, R49. 10.1186/gb-2014-15-3-r49 24594098 PMC4053808

[B196] Wen L. Tang F. (2014). Genomic distribution and possible functions of DNA hydroxymethylation in the brain. Genomics 104, 341–346. 10.1016/j.ygeno.2014.08.020 25205307

[B197] Wijetunga N. A. Johnston A. D. Maekawa R. Delahaye F. Ulahannan N. Kim K. (2017). Smite: An R/bioconductor package that identifies network modules by integrating genomic and epigenomic information. BMC Bioinforma. 18, 41. 10.1186/s12859-017-1477-3 PMC5242055 28100166

[B198] Wnuk A. Rzemieniec J. Litwa E. Lasoń W. Krzeptowski W. Wójtowicz A. K. (2016). The crucial involvement of retinoid X receptors in DDE neurotoxicity. Neurotox. Res. 29, 155–172. 10.1007/s12640-015-9572-6 26563996 PMC4701765

[B199] Wu H. Zhang Y. (2011). Mechanisms and functions of Tet protein-mediated 5-methylcytosine oxidation. Genes Dev. 25, 2436–2452. 10.1101/gad.179184.111 22156206 PMC3243055

[B200] Xu Z. Taylor J. A. Leung Y.-K. Ho S.-M. Niu L. (2016). oxBS-MLE: an efficient method to estimate 5-methylcytosine and 5-hydroxymethylcytosine in paired bisulfite and oxidative bisulfite treated DNA. Bioinformatics 32, btw527–3669. 10.1093/bioinformatics/btw527 PMC5181539 27522082

[B202] Yu M. Han D. Hon G. C. He C. (2018). “Tet-assisted bisulfite sequencing (TAB-seq),” in DNA methylation protocols methods in molecular biology. Editor Tost J. (New York, NY: Springer), 645–663. 10.1007/978-1-4939-7481-8_33 PMC6606048 29224168

[B203] Zhang F. Chen W. Zhu Z. Zhang Q. Nabais M. F. Qi T. (2019). Osca: A tool for omic-data-based complex trait analysis. Genome Biol. 20, 107. 10.1186/s13059-019-1718-z 31138268 PMC6537380

[B204] Zhang H.-Q. Wang J.-Y. Li Z.-F. Cui L. Huang S.-S. Zhu L.-B. (2021). DNA methyltransferase 1 is dysregulated in Parkinson's disease via mediation of miR-17. Mol. Neurobiol. 58, 2620–2633. 10.1007/s12035-021-02298-w 33483902

[B205] Zhang T.-Y. Keown C. L. Wen X. Li J. Vousden D. A. Anacker C. (2018). Environmental enrichment increases transcriptional and epigenetic differentiation between mouse dorsal and ventral dentate gyrus. Nat. Commun. 9, 298. 10.1038/s41467-017-02748-x 29352183 PMC5775256

[B206] Zhang Y. Xu S. Qian Y. He X. Mo C. Yang X. (2022a). Sodium butyrate attenuates rotenone-induced toxicity by activation of autophagy through epigenetically regulating PGC-1α expression in PC12 cells. Brain Res. 1776, 147749. 10.1016/j.brainres.2021.147749 34896331

[B207] Zhang Z. Lee M. K. Perreard L. Kelsey K. T. Christensen B. C. Salas L. A. (2022b). Navigating the hydroxymethylome: Experimental biases and quality control tools for the tandem bisulfite and oxidative bisulfite illumina microarrays. Epigenomics 14, 139–152. 10.2217/epi-2021-0490 35029129 PMC8914583

[B208] Zhao Q. Liu H. Cheng J. Zhu Y. Xiao Q. Bai Y. (2019). Neuroprotective effects of lithium on a chronic MPTP mouse model of Parkinson's disease via regulation of α-synuclein methylation. Mol. Med. Rep. 19, 4989–4997. 10.3892/mmr.2019.10152 31059019

[B209] Zhou J. Broe M. Huang Y. Anderson J. P. Gai W.-P. Milward E. A. (2011). Changes in the solubility and phosphorylation of α-synuclein over the course of Parkinson's disease. Acta Neuropathol. 121, 695–704. 10.1007/s00401-011-0815-1 21400129

[B210] Zhou W. Barkow J. C. Freed C. R. (2017). Running wheel exercise reduces α-synuclein aggregation and improves motor and cognitive function in a transgenic mouse model of Parkinson's disease. PLoS ONE 12, e0190160. 10.1371/journal.pone.0190160 29272304 PMC5741244

[B211] Zocher S. Overall R. W. Lesche M. Dahl A. Kempermann G. (2021). Environmental enrichment preserves a young DNA methylation landscape in the aged mouse hippocampus. Nat. Commun. 12, 3892. 10.1038/s41467-021-23993-1 34162876 PMC8222384

