# Supplementary material for: DNA methylation as a mediator of genetic and environmental influences on Parkinson’s disease susceptibility: Impacts of alpha-Synuclein, physical activity, and pesticide exposure on the epigenome
Source: Front Genet. 2022 Aug 19;13:971298. doi: 10.3389/fgene.2022.971298 (PMC9437223; doi:10.3389/fgene.2022.971298)
Supplement: Supplementary file 1 [file DataSheet1.docx]

**Supplementary Table 1. DNA modification studies in Parkinson’s disease brain tissue**

| **Brain region(s) or cell type(s)** | **Sample size** | **Platform** | **Analysis method** | **Findings** | **Reference** |
| --- | --- | --- | --- | --- | --- |
| Cortex, putamen | *n* = 12  (6 cases,  6 controls) | Illumina 27K array | DiffScore (GenomeStudio), *t* test | ↓ DNAm and ↑ mRNA expression of *CYP2E1* | Kaut *et al*., 2012 |
| Frontal cortex | *n* = 11  (5 cases,  6 controls) | Illumina 450K array | Illumina custom model | 2908 differentially methylated CpGs  Overall ↓ DNAm | Masliah *et al*., 2013 |
| Cerebellum, frontal cortex, pons, temporal cortex | *n* = 15711  (6529 cases,  9452 controls) | Illumina 27K array | Mixed linear model to impute DNAm values from PD GWAS, logistic regression | 6 differentially methylated CpGs | Rawlik *et al*., 2016 |
| Substantia nigra, parietal cortex, occipital cortex | *n* = 10  (5 cases,  5 controls) | Bisulfite pyrosequencing | Mann-Whitney U test | *SNCA* and *PRKN* promoter DNAm changes in all brain regions  *PINK1* promoter DNAm change in substantia nigra | Navarro-Sánchez *et al*., 2018 |
| Cerebellum, brainstem, neocortex | *n* = 16, cerebellum and brainstem  (8 cases,  8 controls)  *n* = 20, neocortex  (10 cases,  10 controls) | Immunohistochemistry | Mann-Whitney U test | ↑ DNAm in PD cortex  ↑ DNAhm in PD cerebellum | Kaut *et al*., 2019 |
| iPSC-derived dopaminergic neurons | *n* = 3  (2 cases,  1 control) | Whole-genome bisulfite sequencing | Inequality of beta distributions (Raineri *et al*., 2014) | Global DNAm increase | Fernández-Santiago *et al*., 2019 |
| Dorsal motor nucleus of the vagus, substantia nigra, cingulate gyrus | *n* = 79  (38 cases,  41 controls) | Illumina 450K array, Illumina EPIC array | Linear regression, comb-p | 234 DMRs in dorsal motor nucleus of the vagus  44 DMRs in substantia nigra  141 DMRs in cingulate gyrus  Enrichment for Wnt signaling | Young *et al*., 2019 |
| Neurons isolated from prefrontal cortex | *n* = 105  (57 cases,  48 controls) | Bisulfite padlock probe sequencing | limma | Greater DNAm hemispheric differences in PD patients than in controls, particularly at CpH sites | Li *et al*., 2020 |
| Neurons isolated from prefrontal cortex | *n* = 105  (57 cases,  48 controls) | Bisulfite padlock probe sequencing | Logistic regression | ↑ DNAhm at enhancers  ↑ *TET2* expression | Marshall *et al*., 2020 |
| Temporal cortex | *n* = 20  (20 cases) | Illumina 450K array | ANCOVA | 7 differentially methylated CpGs related to pesticide exposure | Go *et al*., 2020 |
| Frontal cortex | *n* = 134  (134 cases) | Illumina 450K array | Conditional analysis | Relationship between PD and splicing or PD and expression was conditional on DNAm for 3/11 candidate genes | Kia *et al*., 2021 |
| Cortex | *n* = 24  (14 cases,  10 controls) | Illumina 450K array | *t* test | ↓ DNAm at *CYP2E1* associated with ↑ CYP2E1 protein in brain | Kaut *et al*., 2022 |

CpG, cytosine-phosphate-guanine dinucleotide; CpH, cytosine-phosphate-H dinucleotide where H represents A, C, or T; DMR, differentially methylated region; DNAhm, DNA hydroxymethylation; DNAm, DNA methylation; PD, Parkinson’s disease.

**Supplementary Table 2. DNA modification studies in Parkinson’s disease peripheral tissues**

| **Tissue(s) or cell type(s)** | **Sample size** | **Platform** | **Analysis method** | **Findings** | **Reference** |
| --- | --- | --- | --- | --- | --- |
| Peripheral blood leukocytes | *n* = 11  (5 cases,  6 controls) | Illumina 450K array | Illumina custom model | 3897 differentially methylated CpGs  Overall ↓ DNAm | Masliah *et al*., 2013 |
| Whole blood | *n* = 45  (15 cases with anxiety,  15 cases without anxiety,  15 controls) | Illumina 450K array | Logistic regression | 20 differentially methylated genes (PD vs. control)  17 differentially methylated genes (PD with anxiety vs. PD without anxiety) | Moore *et al*., 2014 |
| Whole blood, saliva | *n* = 572, blood (335 cases, 237 controls)  *n* = 259, saliva (128 cases, 131 controls) | Illumina 450K array | Linear regression, weighted gene coexpression network analysis (WGCNA) | 82 differentially methylated CpGs in blood  5 differentially methylated CpGs in saliva  Immune system, mitochondrial function, cytoskeletal organization, iron handling genes | Chuang *et al*., 2017 |
| Whole blood | *n* = 380  (189 cases,  191 controls) | Illumina EPIC array | limma, DMRcate | 7 differentially methylated CpGs (cross-sectional)  24 differentially methylated regions, including *CYP2E1* (cross-sectional)  138 differentially methylated CpGs (longitudinal) | Henderson-Smith *et al*., 2019 |
| Whole blood | *n* = 572  (335 cases, 237 controls) | Illumina 450K array | limma | 85 genes with ↓ DNAm and ↑ expression in PD | Wang *et al*., 2019 |
| Peripheral blood | *n* = 20  (20 cases) | Illumina 450K array | ANCOVA | 123 differentially methylated CpGs related to pesticide exposure | Go *et al*., 2020 |
| Whole blood | *n* = 2131  (1132 cases,  999 controls) | Illumina 450K array | Mixed linear model (MOMENT, MOA) Summary-based Mendelian randomization | 2 differentially methylated CpGs  ↑ DNAm of cg06690548 associated with ↓ expression of *SLC7A11* | Vallerga *et al*., 2020 |
| Peripheral blood mononuclear cells | *n* = 160  (101 cases,  59 controls) | Bisulfite pyrosequencing | *t* test, multiple linear regression | ↑ DNAm at *DAT1* 5′ UTR | Rubino *et al*., 2020 |
| Whole blood | *n* = 30  (15 cases,  15 controls) | Illumina 450K array | BumpHunter | 31 DMRs (13 with ↑ DNAm, 18 with ↓ DNAm) | Henderson *et al*., 2021 |

CpG, cytosine-phosphate-guanine dinucleotide; CpH, cytosine-phosphate-H dinucleotide where H represents A, C, or T; DMR, differentially methylated region; DNAhm, DNA hydroxymethylation; DNAm, DNA methylation; PD, Parkinson’s disease.

**Supplementary Table 3. Epidemiological and experimental evidence implicating pesticide exposure in Parkinson’s disease etiology**

| **Study type** | **Population or model system** | **Sample size** | **Outcome** | **Findings** | **Reference** |
| --- | --- | --- | --- | --- | --- |
| Observational | Patients with parkinsonism and history of heroin abuse | *n* = 3 cases | Exposure to MPTP (synthetic heroin) | All patients developed parkinsonism within 1 week of heroin use, which persisted for 3–5 days after stopping use | Langston *et al*., 1983 |
| Observational | Patients with early-onset idiopathic PD born and raised in Saskatchewan, Canada | *n* = 22 cases | Well water consumption | 20/22 patients had rural well water exposure in the first 15 years of life  Heavy metal and herbicide/pesticide content of water unrelated to PD | Rajput *et al*., 1987 |
| Epidemiological | Individuals enrolled in Mutualité Sociale Agricole (French health insurance for agricultural workers) | *n* = 781  (224 cases, 557 controls) | Overall pesticide exposure, insecticide/  fungicide/herbicide exposure, exposure to 29 individual pesticides in men | Dose-dependent association between PD and overall pesticide use  In men, dose-dependent association between PD and insecticide/organochlorine use | Elbaz *et al*., 2009 |
| Epidemiological | Private pesticide applicators and their spouses (FAME study, within AHS study) | *n* = 468  (110 cases, 358 controls) | Exposure to pesticides impacting mitochondrial function or oxidative stress | Association between PD and mitochondrial complex I-inhibiting pesticides, e.g., rotenone  Association between PD and oxidative stress-causing pesticides, e.g., paraquat | Tanner *et al*., 2011 |
| Epidemiological | Prospective cohort of active and retired French agricultural workers (AGRICAN study) | *n* = 149 810  (1732 cases,  148 078 controls) | Self-reported exposure to 13 crops and 5 pesticides | Association between PD and overall pesticide use  Association between PD and rotenone, diquat, paraquat, and dithiocarbamates | Pouchieu *et al*., 2018 |
| Experimental | Mouse mesencephalic neurons (dopaminergic, GABAergic) | *n* = 3–9 biological replicates | 5 nM rotenone exposure (6 hours)  100 μM glutamate exposure (1 hour) | NMDA receptor-dependent selective toxicity of dopaminergic neurons to glutamate and rotenone | Marey-Semper *et al*., 1995 |
| Experimental | 2-month old male Lewis rats | *n* = 25 rotenone-treated,  unknown number vehicle-treated | 2–3 mg/kg/day rotenone infusion for 7 days to 5 weeks | Rotenone-treated rats had nigrostriatal lesions, selective dopaminergic degeneration, hypokinesia, rigidity | Betarbet *et al*., 2000 |
| Experimental | Male C57BL/6 mice aged 6 weeks, 8 weeks, 6 months, 18 months | *n* ≥ 4 biological replicates | 3 weekly intraperitoneal paraquat (1–10 mg/kg) or saline injections | Dose- and age-dependent selective vulnerability of dopaminergic neurons to paraquat | McCormack *et al*., 2002 |
| Experimental | Mouse primary mesencephalic neurons | *n* = 4–5 biological replicates | 400 μM paraquat exposure (18–24 hours) | Paraquat-induced phosphorylation of JNK and c-Jun, caspase-3 activation, dopaminergic degeneration  Cell death prevented by JNK inhibitor CEP-11004 | Peng *et al*., 2004 |

JNK, c-Jun N-terminal kinase; MPTP, 1-methyl-4-phenyl-1,2,3,6-tetrahydropyridine; PD, Parkinson’s disease; NDMA, N-methyl-d-aspartate.

**Supplementary Table 4. Selected examples of gene–pesticide exposure interactions in Parkinson’s disease**

| **Study type** | **Population or model system** | **Sample size** | **Outcome(s)** | **Findings** | **Reference** |
| --- | --- | --- | --- | --- | --- |
| Experimental | 6–8-month-old male *LRRK2* knockout or transgenic *LRRK2* G2019S mutant mice | *n* = 48 | 10 mg/kg paraquat or saline injection every other day for 6 days  Signs of sickness, inflammation, and home-cage activity | *LRRK2* knockout prevented paraquat-induced sickness, inflammation, and motivation/activity deficits  *LRRK2* G2019S associated with elevated corticosterone | Rudyk *et al*., 2019 |
| Association | Patients and controls recruited from three California, USA counties | *n* = 658  (324 cases,  334 controls) | Occupational pesticide exposure  Residential maneb and paraquat exposure  *DAT* genotype | Dose-dependent association between PD risk and *DAT* genotype  Joint impact of *DAT* genotype and paraquat, maneb, or occupational pesticide exposure on PD risk | Ritz *et al*., 2009 |
| Association | Individuals enrolled in Mutualité Sociale Agricole (French health insurance for agricultural workers) | *n* = 923  (247 cases,  676 controls) | Occupational pesticide exposure  *CYP2D6* genotype | Joint impact of *CYP2D6* genotype and occupational pesticide exposure on PD risk | Elbaz *et al*., 2004 |
| Association | Caucasian individuals of Russian descent recruited from St. Petersburg, Russia | *n* = 1230 | *CYP2D6* genotype  *CP2D6* DNA methylation  Smoking behavior | Age, sex, and genotype influenced *CYP2D6* DNA methylation levels  Higher *CYP2D6* DNA methylation associated with smoking behavior | Tiili *et al*., 2015 |

PD, Parkinson’s disease.

**Supplementary Table 5. Epidemiological and experimental evidence for preventive effect of physical activity on Parkinson’s disease**

| **Study type** | **Population or model system** | **Sample size** | **Outcome(s)** | **Findings** | **Reference** |
| --- | --- | --- | --- | --- | --- |
| Prospective cohort | Male students attending University of Pennsylvania or Harvard College between 1916 and 1950 | *n* = 50 002  (164 cases,  4 controls selected per case) | Physical activity during college and adulthood | College varsity sports or exercise associated with lower risk of PD later in life  Adulthood physical activity associated with lower PD risk | Sasco *et al*., 1992 |
| Prospective cohort | Health Professionals Follow-Up Study (HPFS), Nurses’ Health Study (NHS) | *n* = 125 828  (387 cases) | Baseline physical activity, strenuous exercise in early adulthood | Physical activity associated with lower PD risk in men  Strenuous exercise associated with lower PD risk in men and women | Chen *et al*., 2005 |
| Prospective cohort | Swedish National March cohort | *n* = 43 368  (286 cases) | Baseline physical activity | Individuals with > 6 hours/week household and commuting activity had lower PD risk  Men with higher physical activity levels had lower PD risk | Yang *et al*., 2015 |
| Randomized controlled trial | PD patients with disease duration ≤ 3 years, Hoehn and Yahr stage 1–2 | *n* = 30 cases | High-intensity treadmill exercise, low-intensity aerobic exercise, zero-intensity | High-intensity exercise increased motor performance and decreased corticomotor excitability | Fisher *et al*., 2008 |
| Randomized controlled trial | PD patients with mild to moderate idiopathic disease | *n* = 96 cases | Treadmill exercise, tango, or stretching | Treadmill exercise increased forward/backward walking velocity | Rawson *et al*., 2019 |
| Experimental | 6- and 8-week-old male and female C57BL/6 mice | *n* = 10–14 mice per condition | Housing in EE | ↓ Global hippocampal DNAhm with EE  Improved learning and memory with EE in aged mice  Altered DNAhm with EE at gene bodies of axon guidance genes | Irier *et al*., 2014 |
| Experimental | 10-month-old male and female wild-type and *DJ-1* knockout C5B7BL/6 mice  15-month-old male and female wild-type and *SNCA* transgenic C57BL/6 mice | *n* = 5–7 mice per condition | 1 week or 3 months of running wheel exercise | *DJ-1* knockout reduced running wheel and rotarod performance  Exercise reduced α-Syn aggregation | Zhou *et al*., 2017 |
| Experimental | 12-month-old female wild-type and *SNCA* transgenic C57BL/6 mice | *n* = 4 mice per condition | *SNCA* genotype  Housing in EE | EE prevented *SNCA*-induced changes to hippocampal transcriptome | Wassouf *et al*., 2018 |
| Experimental | 80-day-old male C57BL/6 mice | *n* = 10–12 mice per condition | Housing in EE | EE increased DNAm and mRNA expression differences between dorsal and ventral dentate gyrus  EE promoted hippocampal neurogenesis | Zhang *et al*., 2018 |
| Experimental | 6.5-week-old and 14-month-old female C57BL/6 mice | *n* = 3–10 mice per condition | Housing in EE | EE prevented age-related hippocampal DNAm changes | Zocher *et al*., 2021 |
| Experimental | P51 female C57BL/6 mice | *n* = 2 mice per condition | Housing in EE | EE increased chromatin accessibility at enhancers and promoters in the cortex  EE altered chromosomal contacts, mRNA/protein expression, histone PTMs | Espeso-Gil *et al*., 2021 |

α-Syn, alpha-synuclein; DNAhm, DNA hydroxymethylation; DNAm, DNA methylation; EE, enriched environment; PD, Parkinson’s disease; PTMs, posttranslational modifications.

**References**

Betarbet, R., Sherer, T. B., MacKenzie, G., Garcia-Osuna, M., Panov, A. V., and Greenamyre, J. T. (2000). Chronic systemic pesticide exposure reproduces features of Parkinson’s disease. *Nat. Neurosci.* 3, 1301–1306. doi: 10.1038/81834.

Chen, H., Zhang, S. M., Schwarzchild, M. A., Hernán, M. A., and Ascherio, A. (2005). Physical activity and the risk of Parkinson disease. *Neurology* 64, 664–669. doi: 10.1212/01.WNL.0000151960.28687.93.

Chuang, Y.-H., Paul, K. C., Bronstein, J. M., Bordelon, Y., Horvath, S., and Ritz, B. (2017). Parkinson’s disease is associated with DNA methylation levels in human blood and saliva. *Genome Med.* 9, 76. doi: 10.1186/s13073-017-0466-5.

Elbaz, A., Levecque, C., Clavel, J., Vidal, J. S., Richard, F., Amouyel, P., *et al.* (2004). CYP2D6 polymorphism, pesticide exposure, and Parkinson’s disease. *Ann. Neurol.* 55, 430–434. doi: 10.1002/ana.20051.

Elbaz, A., Clavel, J., Rathouz, P. J., Moisan, F., Galanaud, J.-P., Delemotte, B., *et al.* (2009). Professional exposure to pesticides and Parkinson disease. *Ann. Neurol.* 66, 494–504. doi: 10.1002/ana.21717.

Espeso-Gil, S., Holik, A. Z., Bonnin, S., Jhanwar, S., Chandrasekaran, S., Pique-Regi, R., *et al.* (2021). Environmental enrichment induces epigenomic and genome organization changes relevant for cognition. *Front. Mol. Neurosci.* 14, 664912. doi: 10.3389/fnmol.2021.664912.

Fernández-Santiago, R., Merkel, A., Castellano, G., Heath, S., Raya, Á., Tolosa, E., *et al.* (2019). Whole-genome DNA hyper-methylation in iPSC-derived dopaminergic neurons from Parkinson’s disease patients. *Clin. Epigenetics* 11, 108. doi: 10.1186/s13148-019-0701-6.

Fisher, B. E., Wu, A. D., Salem, G. J., Song, J., Lin, C. H. J., Yip, J., *et al.* (2008). The effect of exercise training in improving motor performance and corticomotor excitability in people with early Parkinson’s disease. *Arch. Phys. Med. Rehabil.* 89, 1221–1229. doi: 10.1016/j.apmr.2008.01.013.

Go, R. C. P., Corley, M. J., Ross, G. W., Petrovitch, H., Masaki, K. H., Maunakea, A. K., *et al.* (2020). Genome-wide epigenetic analyses in Japanese immigrant plantation workers with Parkinson’s disease and exposure to organochlorines reveal possible involvement of glial genes and pathways involved in neurotoxicity. *BMC Neurosci.* 21, 31. doi: 10.1186/s12868-020-00582-4.

Henderson, A. R., Wang, Q., Meechoovet, B., Siniard, A. L., Naymik, M., De Both, M., *et al.* (2021). DNA methylation and expression profiles of whole blood in Parkinson’s disease. *Front. Genet.* 12, 640266. doi: 10.3389/fgene.2021.640266.

Henderson-Smith, A., Fisch, K. M., Hua, J., Liu, G., Ricciardelli, E., Jepsen, K., *et al.* (2019). DNA methylation changes associated with Parkinson’s disease progression: outcomes from the first longitudinal genome-wide methylation analysis in blood. *Epigenetics* 14, 365–382. doi: 10.1080/15592294.2019.1588682.

Irier, H., Street, R. C., Dave, R., Lin, L., Cai, C., Davis, T. H., *et al.* (2014). Environmental enrichment modulates 5-hydroxymethylcytosine dynamics in hippocampus. *Genomics* 104, 376–382. doi: 10.1016/j.ygeno.2014.08.019.

Kaut, O., Kuchelmeister, K., Moehl, C., and Wüllner, U. (2019). 5-Methylcytosine and 5-hydroxymethylcytosine in brains of patients with multiple system atrophy and patients with Parkinson’s disease. *J. Chem. Neuroanat.* 96, 41–48. doi: 10.1016/j.jchemneu.2018.12.005.

Kaut, O., Schmitt, I., and Wüllner, U. (2012). Genome-scale methylation analysis of Parkinson’s disease patients’ brains reveals DNA hypomethylation and increased mRNA expression of cytochrome P450 2E1. *Neurogenetics* 13, 87–91. doi: 10.1007/s10048-011-0308-3.

Kaut, O., Schmitt, I., Stahl, F., Fröhlich, H., Hoffmann, P., Gonzalez, F. J., Wüllner, U. (2022). Epigenome-Wide Analysis of DNA Methylation in Parkinson's Disease Cortex. *Life (Basel)* 12, 502. doi: 10.3390/life12040502.

Kia, D. A., Zhang, D., Guelfi, S., Manzoni, C., Hubbard, L., Reynolds, R. H., *et al.* (2021). Identification of candidate Parkinson disease genes by integrating genome-wide association study, expression, and epigenetic data sets. *JAMA Neurol.* 78, 464–472. doi: 10.1001/jamaneurol.2020.5257.

Langston, J. W., Ballard, P., Tetrud, J. W., and Irwin, I. (1983). Chronic parkinsonism in humans due to a product of meperidine-analog synthesis. *Science* 219, 979–980. doi: 10.1126/science.6823561.

Li, P., Ensink, E., Lang, S., Marshall, L., Schilthuis, M., Lamp, J., *et al.* (2020). Hemispheric asymmetry in the human brain and in Parkinson’s disease is linked to divergent epigenetic patterns in neurons. *Genome Biol*. 21, 61. doi: 10.1186/s13059-020-01960-1.

Marey-Semper, I., Gelman, M., and Lévi-Strauss, M. (1995). A selective toxicity toward cultured mesencephalic dopaminergic neurons is induced by the synergistic effects of energetic metabolism impairment and NMDA receptor activation. *J. Neurosci.* 15, 5912–5918. doi: 10.1523/jneurosci.15-09-05912.1995.

Marshall, L. L., Killinger, B. A., Ensink, E., Li, P., Li, K. X., Cui, W., *et al.* (2020). Epigenomic analysis of Parkinson’s disease neurons identifies Tet2 loss as neuroprotective. *Nat. Neurosci*. 23, 1203–1214. doi: 10.1038/s41593-020-0690-y.

Masliah, E., Dumaop, W., Galasko, D., and Desplats, P. (2013). Distinctive patterns of DNA methylation associated with Parkinson disease: identification of concordant epigenetic changes in brain and peripheral blood leukocytes. *Epigenetics* 8, 1030–1038. doi: 10.4161/epi.25865.

McCormack, A. L., Thiruchelvam, M., Manning-Bog, A. B., Thiffault, C., Langston, J. W., Cory-Slechta, D. A., *et al.* (2002). Environmental risk factors and Parkinson’s disease: selective degeneration of nigral dopaminergic neurons caused by the herbicide paraquat. *Neurobiol. Dis.* 10, 119–127. doi: 10.1006/nbdi.2002.0507.

Moore, K., McKnight, A. J., Craig, D., and O’Neill, F. (2014). Epigenome-wide association study for Parkinson’s disease. *Neuromol. Med*. 16, 845–855. doi: 10.1007/s12017-014-8332-8.

﻿Navarro-Sánchez, L., Águeda-Gómez, B., Aparicio, S., and Pérez-Tur, J. (2018). ﻿Epigenetic Study in Parkinson's Disease: A Pilot Analysis of DNA Methylation in Candidate Genes in Brain. *Cells* 7, 150. doi: ﻿10.3390/CELLS7100150

Peng, J., Mao, X. O., Stevenson, F. F., Hsu, M., and Andersen, J. K. (2004). The herbicide paraquat induces dopaminergic nigral apoptosis through sustained activation of the JNK pathway. *J. Biol. Chem.* 279, 32626–32632. doi: 10.1074/jbc.M404596200.

Pouchieu, C., Piel, C., Carles, C., Gruber, A., Helmer, C., Tual, S., *et al.* (2018). Pesticide use in agriculture and Parkinson’s disease in the AGRICAN cohort study. *Int. J. Epidemiol.* 47, 299–310. doi: 10.1093/ije/dyx225.

Rajput, A. H., Uitti, R. J., Stern, W., Laverty, W., O’Donnell, K., O’Donnell, D., *et al.* (1987). Geography, drinking water chemistry, pesticides and herbicides and the etiology of Parkinson’s disease. *Can. J. Neurosci.* 14, 414–418. doi: 10.1017/s0317167100037823.

Rawlik, K., Rowlatt, A., and Tenesa, A. (2016). Imputation of DNA methylation levels in the brain implicates a risk factor for Parkinson’s disease. *Genetics* 204, 771–781. doi: 10.1534/genetics.115.185967.

Rawson, K. S., McNeely, M. E., Duncan, R. P., Pickett, K. A., Perlmutter, J. S., and Earhart, G. M. (2019). Exercise and Parkinson disease: comparing tango, treadmill, and stretching. *J. Neurol. Phys. Ther.* 43, 26–32. doi: 10.1097/NPT.0000000000000245.

Ritz, B. R., Manthripragada, A. D., Costello, S., Lincoln, S. J., Farrer, M. J., Cockburn, M., and Bronstein, J. (2009). Dopamine Transporter Genetic Variants and Pesticides in Parkinson’s Disease. *Environ. Health Persp.* 117, 964–969. doi: 10.1289/ehp.0800277.

Rubino, A., D’Addario, C., Di Bartolomeo, M., Michele Salamone, E., Locuratolo, N., Fattapposta, F., *et al.* (2020). DNA methylation of the 5′-UTR DAT 1 gene in Parkinson’s disease patients. *Acta Neurol. Scand.* 142, 275–280. doi: 10.1111/ane.13279.

Rudyk, C., Dwyer, Z., Hayley, S., and CLINT membership (2019). Leucine-rich repeat kinase-2 (LRRK2) modulates paraquat-induced inflammatory sickness and stress phenotype. *J Neuroinflammation* 16, 120. doi: 10.1186/s12974-019-1483-7.

Sasco, A. J., Paffenbarger, R. S., Gendre, I., and Wing, A. L. (1992). The role of physical exercise in the occurrence of Parkinson’s disease. *Arch. Neurol.* 49, 360–365. doi: [10.1001/archneur.1992.00530280040020](https://doi.org/10.1001/archneur.1992.00530280040020).

Tanner, C. M., Kamel, F., Ross, G. W., Hoppin, J. A., Goldman, S. M., Korell, M., *et al.* (2011). Rotenone, paraquat, and Parkinson’s disease. *Environ. Health Perspect.* 119, 866–872. doi: 10.1289/ehp.1002839.

Tiili, E. M., Antikainen, M. S. H., Mitiushkina, N. V., Sukhovskaya, O. A., Imyanitov, E. N., and Hirvonen, A. P. (2015). Effect of genotype and methylation of *CYP2D6* on smoking behaviour. *Pharmacogenet. Genomics* 25, 531–540. doi: 10.1097/FPC.0000000000000166.

Vallerga, C. L., Zhang, F., Fowdar, J., McRae, A. F., Qi, T., Nabais, M. F., *et al.* (2020). Analysis of DNA methylation associates the cystine–glutamate antiporter SLC7A11 with risk of Parkinson’s disease. *Nat. Commun.* 11, 1238. doi: 10.1038/s41467-020-15065-7.

Yang, F., Trolle Lagerros, Y., Bellocco, R., Adami, H. O., Fang, F., Pedersen. N. L., and Wirdefeldt, K. (2015). Physical activity and risk of Parkinson’s disease in the Swedish National March Cohort. *Brain* 138, 269–275. doi: 10.1093/brain/awu323.

﻿Young, J. I., Sivasankaran, S. K., Wang, L., Ali, A., Mehta, A., Davis, D. A., *et al.* (2019). ﻿Genome-wide brain DNA methylation analysis suggests epigenetic reprogramming in Parkinson disease. *Neurol. Genet.* 5, e342. doi: ﻿10.1212/NXG.0000000000000342

Wang, C., Chen, L., Yang, Y., Zhang, M., and Wong, G. (2019). Identification of potential blood biomarkers for Parkinson’s disease by gene expression and DNA methylation data integration analysis. *Clin. Epigenet.* 11, 24. doi: 10.1186/s13148-019-0621-5.

Wassouf, Z., Hentrich, T., Samer, S., Rotermund, C., Kahle, P. J., Ehrlich, I., *et al.* (2018). Environmental enrichment prevents transcriptional disturbances induced by alpha-synuclein overexpression. *Front. Cell Neurosci.* 12, 112. doi: 10.3389/fncel.2018.00112.

Zhang, T.-Y., Keown, C. L., Wen, X., Li, J., Vousden, D. A., Anacker, C., *et al.* (2018). Environmental enrichment increases transcriptional and epigenetic differentiation between mouse dorsal and ventral dentate gyrus. *Nat. Commun.* 9, 298. doi: 10.1038/s41467-017-02748-x.

Zhou, W., Barkow, J. C., and Freed, C. R. (2017). Running wheel exercise reduces α-synuclein aggregation and improves motor and cognitive function in a transgenic mouse model of Parkinson’s disease. *PLoS ONE* 12, e0190160. doi: 10.1371/journal.pone.0190160.

Zocher, S., Overall, R. W., Lesche, M., Dahl, A., and Kempermann, G. (2021). Environmental enrichment preserves a young DNA methylation landscape in the aged mouse hippocampus. *Nat. Commun.* 12, 3892. doi: 10.1038/s41467-021-23993-1.
